# Understanding Human Motion from Depth Sensors: Activity Recognition and Age Group Recognition Using Skeleton Data

**DOI:** 10.3390/s26175453

**Published:** 2026-08-28

**Authors:** Rinu Elizabeth Paul, Alp Göktug Tanman, Yale Hartmann, Jordan Behrendt, Hui Liu, Tanja Schultz

**Affiliations:** Cognitive Systems Lab, University of Bremen, Enrique-Schmidt-Str. 5, 28359 Bremen, Germanyjobe@uni-bremen.de (J.B.);

**Keywords:** older adults, activity recognition, age group recognition, depth sensor, skeleton joints, nursing homes, elderly monitoring

## Abstract

Human Activity Recognition (HAR) plays a significant role in various applications, from learning a discipline to physical rehabilitation. In older adults, activity patterns can indicate levels of frailty, which helps inform the design of physical training programs to prevent falls and maintain mobility. HAR sensing ranges from wearable sensors such as IMUs and RGB cameras to video, specialized gait laboratories, perturbation units, VR, and other modalities. This paper presents a comprehensive study of depth-based, skeleton-driven HAR and age group recognition (AGR) using data collected from real-world nursing home environments. Depth sensors offer a privacy-preserving and non-invasive alternative to wearable and RGB-based systems, enabling continuous 24-h monitoring without requiring user compliance. We systematically evaluate multiple modeling paradigms, including classical machine learning models (DT, RF, KNN, SVM, HMM, HMM+SVM), sequence-based models (LSTM, TCN, ARNN), and graph-based approaches, using skeletal joint data extracted from depth images. Experiments are conducted on two heterogeneous datasets: NTU RGB+D (younger adults) and ETAP-DID (older adults). We analyze the impact of different joint subset configurations (full-body, limb-only, leg-only, and torso-only) and compare raw joint representations with handcrafted time-series features (TSFEL) for frame-based HAR. Beyond activity recognition, we introduce an AGR pipeline to distinguish younger from older adults based on skeletal motion patterns. We investigate multiple feature representations, including absolute joint positions, root-relative coordinates, bone vectors, and joint velocities, and provide interpretability through feature importance and saliency analysis to identify age-discriminative joints and motion cues. Our study provides a comprehensive analysis of various HAR models applied to depth data, examining model performance and the contribution of joint-based features to HAR and AGR. Our study highlights the potential for personalized privacy-preserved monitoring and intervention in nursing homes.

## 1. Introduction

Human Activity Recognition (HAR) is crucial for recognizing activities of daily living (ADLs). HAR applications fall into broad categories: Healthcare, Human–Computer Interaction (HCI), smart environments, safety and security, and sports [1]. Some applications in these categories include fall detection, rehabilitation, action control, adaptive User Interface (UI), IoT monitoring, energy management, surveillance, industrial safety monitoring, coaching, and fitness trackers. The data for HAR are collected using various sensors that fall into two categories: wearable Inertial Measurement Units (IMUs) [2], which include accelerometers and gyroscopes placed on the human body, and fixed vision-based sensors [3], such as cameras and smartphones, placed at a distance from humans. HAR applies to younger and older adults. For younger populations (age below 65), potential applications of HAR include sports training, action-related UI, industrial safety monitoring, and more. For older adults (aged 65 and older), key applications include fall detection, movement monitoring, mobility assessment, and more.

### 1.1. Motivation

HAR can play an important role in nursing homes by improving care and enabling faster, more accurate detection of motor-related diseases. There are various applications of HAR in a nursing home. Falls are a critical concern for adults over 65; fall prevention is an important area of focus [4]. Frailty of an individual and fall risk probability can be measured using mobility and balance assessment tests such as Tinetti in [5,6], Timed Up and Go (TUG) in [6,7,8,9], and Short Physical Performance Battery (SPPB) in [10], which are widely used in geriatric medicine and nursing care. Continuous monitoring of nursing home residents and automated assessments to predict frailty can help establish precautions to ensure individual safety. Activity monitoring, gait analysis, and mobility assessment are frailty indicators and are beneficial for the early detection of gait-based diseases such as Parkinson’s disease [11,12]. Sleep position monitoring is useful for bedridden adults, as it can track position changes and durations, providing caregivers with indications of these changes and helping prevent bed sores [13]. The bed exit model can predict an exit by monitoring a series of activities beforehand, thereby notifying the caregiver and preventing a fall. Bed-exit detection is advantageous for older adults who often fall when standing up from the bed due to balance issues or medication side effects, but who may forget to call a caregiver for assistance.

Wearable sensors provide the best estimate of human activity, but they are impractical for 24/7 monitoring. For continuous monitoring, battery life and electrode degradation pose the main challenges, necessitating periodic replacement and rendering continuous monitoring impractical [14]. Furthermore, continuous use of wearable sensors can be uncomfortable due to skin irritation. Water and outdoor exposure may cause damage and shorten the sensor’s lifespan. Another key factor regarding wearables for older adults is that they often forget to wear the sensors. A new category is hearables, which support hearing and are recently used for HAR purposes [15]. Vision-based sensors, such as cameras, can be used for continuous monitoring. These can be mounted in a fixed location and supplied with continuous power. These can be placed in individual rooms and common areas. The main limitations of this approach are its narrow field of view (FOV) and privacy concerns with RGB cameras [16]. Limited FOV can be addressed to some extent with multiple cameras. Privacy issues can be addressed by using depth cameras, which preserve privacy by generating a structure of objects and subjects within the FOV rather than by recognizing person-specific features such as faces. Depth cameras are a practical, non-wearable, non-invasive, and privacy-preserving approach.

While most studies on depth are based on younger adults, Van der Kruk et al. [17] investigate biomechanical differences in movement between older and younger adults, highlighting how aging influences compensatory strategies during a common functional task. Their study reveals that older adults adopt altered movement patterns, such as modified timing and joint coordination, to maintain stability and compensate for age-related declines in strength and balance. Activity performed by older adults and younger adults highlights significant differences [17,18]. Mobility declines with age, as shown by the movement pattern. Hence, activity recognition models and analyses for both younger and older adults are equally important.

When this difference warrants age-group-specific HAR, it could help differentiate between age groups. Age group recognition is helpful when the application requires separating or focusing on a specific age group. Recognizing children or older adults in a crowded public or semi-public environment can be achieved quickly using age group recognition. In these scenarios, automated age group recognition (AGR) is beneficial for target monitoring, safety, and assistance. In nursing homes and assisted living facilities, older adults are often accompanied and cared for by care staff. For an older adult-focused movement tracking, it is crucial to separate older adults from the care personnel. This monitoring can detect mobility, deviations in movement patterns, early signs of motor decline, and resident wandering. These applications and scenarios make AGR relevant in real-world settings with diverse age groups.

### 1.2. State of the Art

Numerous studies on HAR have employed diverse approaches and algorithms across sensor types, data collection sites, and recognition methods. Here, we give an overview of work on HAR and AGR from which we borrowed ideas. We readily identify the research gaps addressed in our work.

#### 1.2.1. Wearable-Based Human Activity Recognition

In [19], 20 subjects (13 males, seven females) performed 20 everyday activities without specific instructions on where or how to carry them out. Participants performed the activities in a semi-naturalistic setup that allowed greater freedom and in a controlled laboratory environment where subjects followed predefined sequences. Data were collected using five wireless biaxial accelerometers (ADXL210E) placed on the right hip, dominant wrist, non-dominant upper arm, dominant ankle, and non-dominant thigh. Features were computed with a 512-sample sliding window with 256-sample overlaps, enabling efficient Fast Fourier Transform (FFT) computation. Extracted features included mean, energy, frequency domain, and correlation. They evaluated the classifiers using Decision Table, Instance-Based Learning, C4.5 Decision Tree (DT), and Naïve Bayes. The C4.5 DT achieved the highest accuracy at 84%.

In [20], data were collected using motion-sensor-based (accelerometers and gyroscopes), with an IMU sensor. The sensor was mounted on the chest to collect motion data. A Kalman Filter was employed to estimate errors in position and velocity over time, assuming a correlation between them. This approach utilizes a Zero Velocity Update (ZVU) method to minimize estimation errors. The motion estimation involved four steps: zero-velocity detection, sit-down/stand-up estimation, lay-down/stand-up estimation, and falling-down estimation. The ZVU criteria improved accuracy in detecting these transitions by leveraging the chest-mounted sensor, which reduced the estimated distance and filtered out large volumes of unnecessary data.

In another study [21], the authors evaluated 10 machine learning (ML) classifiers on three datasets—PAMAP2, MHEALTH, and SWELL—using performance and runtime metrics. Due to hardware constraints and prolonged runtimes in various models, neural network-based techniques were excluded. The datasets differed in sensor types (e.g., ECG (Electrocardiograph) sensor and linear acceleration sensor), sampling frequencies (50 Hz vs. 100 Hz), activity environments, and attribute sets. Activities were analyzed as individual activities (e.g., sitting, standing, running), grouped activities (locomotive vs. stationary), and common activities across all datasets (e.g., walking, sitting, running, cycling, and climbing stairs). It was observed that grouped activities performed better. Among the evaluated classifiers, the DT with Entropy outperformed others in both performance metrics and runtime.

Smartphone inertial sensors were used for HAR, with a focus on fall detection using the UNIBA dataset [22]. Data were collected from smartphone accelerometers and gyroscopes positioned in the user’s front trouser pocket. A total of 20 subjects participated, performing daily activities and simulated falls under controlled conditions. The dataset included six classes: walking, standing, sitting, lying down, falling, and transitions. Data were segmented into fixed-length windows, and time- and frequency-domain features were extracted. Several ML algorithms were evaluated, including SVM, k-Nearest Neighbors (KNNs), and Random Forest (RF). Among these, RF achieved the highest performance, with over 98% accuracy in fall recognition. The study demonstrates the effectiveness of smartphone-based HAR systems in accurately detecting falls using a minimal sensor setup and a robust classification pipeline.

#### 1.2.2. Camera-Based Human Activity Recognition

In camera-based HAR, pose estimation is first applied to images to extract the joint positions (skeletal joints) of the human present in the image. Skeleton extraction is beneficial for models, as it focuses on body movement rather than full information, such as backgrounds and clothing, leading to more efficient, robust, and scalable models.

Support Vector Machine (SVM) was used for classification due to its effectiveness in handling high-dimensional feature spaces, as reported in [23]. Skeleton joint data from 20 joints were extracted using a pose estimation from a Kinect sensor. Three body positions—standing, sitting, and lying down—were used to animate eight activities, including drinking, playing on a phone, reading, watching TV, and sleeping. The evaluation was conducted at three subject-to-camera distances (one, two, and three meters). The best performance, 93.75% accuracy, was achieved at a distance of two meters.

A hybrid model combining SVM and Hidden Markov Model (HMM) was proposed in [24] to leverage the strengths of both. SVM for multi-class classification and HMM for temporal pattern recognition. The approach involved independent training of both models, followed by an additional training phase for the HMM using multiple observation sequences. Three-dimensional skeletal data were collected using a Kinect sensor. Polar features (distance from the center joint to 14 other joints), motion features (3D coordinates across two adjacent frames for 15 joints), and spatial structure features (relationships between joint pairs) were extracted. The model was trained to classify 13 activities, including brushing teeth, wearing contact lenses, and writing on a whiteboard. It was evaluated on both seen and unseen subjects, achieving an accuracy of 98.22%, outperforming the standalone SVM (94.9%) and HMM (93.58%) models.

#### 1.2.3. Age Group Recognition

AGR using gait analysis is a well-studied topic across different age groups and genders. In [25], a gait database was used for multi-view classification of subjects’ age and gender with children (4–15 years), adult males (15–65 years), adult females (15–65 years), and older adults (65+ years). The setup was a treadmill surrounded by 25 synchronized Video Graphics Array (VGA) cameras and six screens, capturing data at 60 frames per second (fps). A total of 168 participants (88 males, 80 females) aged between 4 and 75 years walked on a treadmill and on the ground. Gait Silhouette Volume (GSV) was used to extract 2D spatial features capturing both static and dynamic characteristics. A KNN algorithm with k = 3 was applied in Linear Discriminant Analysis (LDA) space, and the Correct Classification Rate was averaged over 20 trials. The classification accuracy reached 80% for adult males and females, and 74% for children and older adults. However, when the age range was limited to 25–34 years, accuracy improved to 91% for adult males and females and to 94% for the children and older adult classification.

In [26], the largest inertial sensor dataset to date was used for age estimation and gender classification. Data were collected from 744 subjects (388 males, 356 females) aged 2–78 years using IMUs with triaxial accelerometers and gyroscopes, placed on both sides of the waist and at the center back. The process consisted of gait cycle identification, feature extraction, and regression/classification. Gait cycles were identified through segmentation and interpolation, and features were extracted using MATLAB. For classification and regression, several models were tested using Weka, including Naïve Bayes (for gender), Logistic Regression, Multi-Layer Perceptron, SVM, and RF. Logistic Regression performed the best for gender classification, with an accuracy of around 68%, followed by RF. SVM performed best with the highest correlation coefficient and the lowest MAE and RMSE for AGR.

### 1.3. Research Gap and Paper Objectives

While HAR has been extensively studied, most existing work focuses on classifying activity types despite the observed differences among them. In particular, the role of skeletal joint information and the contributions of specific joints to activity recognition and age discrimination remain underexplored. Moreover, HAR systems typically rely on RGB cameras or wearable sensors, which either raise privacy concerns or require active user participation, thereby limiting their suitability for continuous monitoring in nursing home settings. In our view, depth sensors offer a privacy-preserving alternative for 24-h monitoring. Yet they have received limited attention in age-based movement analyses of datasets collected in shared real-world environments, such as nursing homes. Further, existing studies rarely investigate which joint-based motion characteristics best indicate age differences, thereby limiting the interpretability and clinical relevance of HAR models. Finally, there is a lack of unified benchmarking across classical ML, temporal, and graph-based models using consistent skeletal representations, making it difficult to draw systematic conclusions about model behavior and generalization.

The gaps motivate the need for depth-based, interpretable HAR frameworks that systematically analyze joint contributions, enable age discrimination from skeletal motion, and provide comprehensive benchmarking across modeling paradigms, supporting personalized monitoring and preventive strategies for older adult care. Therefore, we focus on the following research questions in this work.

Human Activity Recognition:

- What are the comparative performances (correctness and computational) of different ML algorithms for HAR using depth-image-based skeleton data?
- What is the impact of raw joint features versus handcrafted time-series features (TSFEL) on frame-based HAR models?
- What is the impact of body parts (all joints, limbs only, legs only, non-limb joints) on frame-based and sequence-based HAR performance using depth-derived skeleton data?

Age Group Recognition:

- Can skeletal motion patterns discriminate between younger and older adults?
- What is the impact of feature representations (positions, velocities, bones, root-relative) that are most age-informative?
- What is the impact of body parts that contribute most to age discrimination for frame-based, sequence-based, and graph-based models for AGR?

To answer the research question, we present the first systematic study of joint subset strategies for skeleton-based HAR, comparing all-joint, leg-only, limb-only, and torso-only configurations. We benchmark six frame-based ML models (DT, RF, KNN, SVM, HMM, HMM+SVM) and three sequence models (LSTM, TCN, ARNN) on two heterogeneous HAR datasets. We perform experiments using raw joint features and TSFEL features with frame-based HAR models. We present an AGR pipeline that uses skeletal data and compares absolute, root-relative, bone-length, and velocity representations. We analyze feature importance and provide interpretability using RF importance and gradient-based saliency techniques for LSTM and ST-GCN. We report insights on which body joints and motion cues are discriminative for HAR and age-based movement analysis.

This paper is further organized as follows: first, depth datasets and how we used them in our experiments; then, the HAR section, which compares the selected models across different joint subsets. The AGR section presents experiments using different input representations, followed by a conclusion and discussion of future work.

## 2. Datasets, Annotation and Feature Extraction

### 2.1. Datasets

To evaluate and develop depth-based HAR models, we used two datasets. These include one collected from nursing home residents, the ETAP-Depth Image Dataset (ETAP-DID), and a widely used public benchmark dataset, NTU RGB+D 120. In both datasets, we focused on the depth data. The depth sensor captured multiple images per second, creating a continuous video stream in which each pixel represented the distance from the sensor. We also explain how the datasets were synchronized in this work to enable more meaningful comparisons.

#### 2.1.1. ETAP-Depth Image Dataset

**Figure 1:**
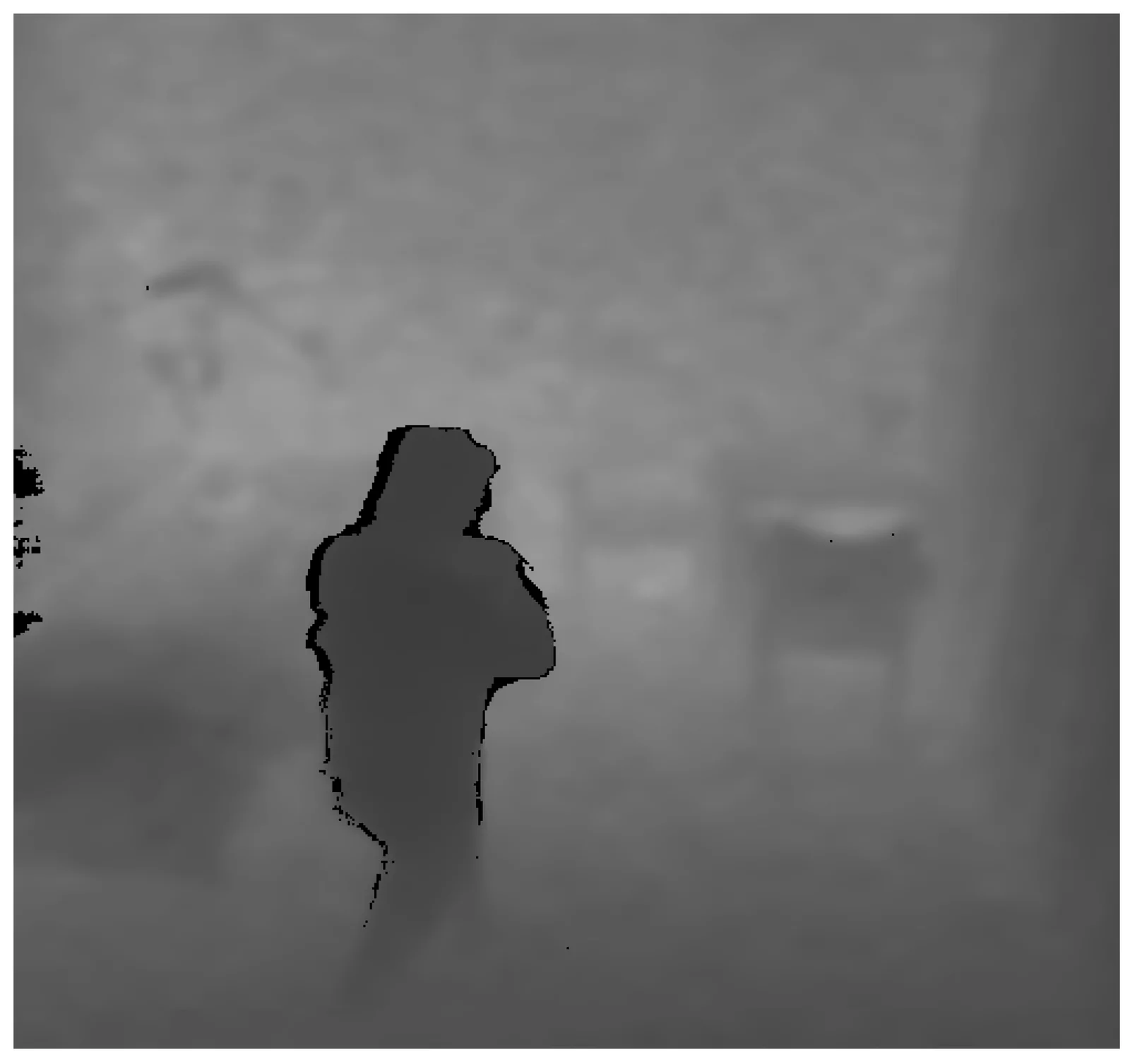
Depth image 640 × 480 showing a resident in the room of a nursing home.

Our dataset, ETAP-DID [27], was collected using sensor boxes developed by DHC Digital HealthCare Systems GmbH that integrated a privacy-preserving depth sensor. The dataset was collected over 21 months from three nursing homes using wall-mounted depth sensors, with one sensor per resident room. The resident room is equipped with a bed, table, sofa, and wardrobe. The bathroom was excluded from recording. It contains 58,037 h of 16-bit depth data from 45 residents aged 60 years and older, recorded at 10 fps with a resolution of 640 × 480 pixels. Depth sensors were mounted approximately 2 m above the ground at a 20° angle to capture a full-body view, with placement optimized for safety, unobtrusiveness, and visibility. Figure 1 presents a sample depth frame from a nursing home. The dataset covered daily activities of older adults, such as sitting, standing, lying, walking, and transition activities (sit-to-stand and vice versa). Here, we focused on single-person scenarios for HAR models and person–staff combination scenarios for the AGR.

#### 2.1.2. NTU RGB+D Dataset

The NTU RGB+D 120 dataset is an extension of the NTU RGB+D dataset, and a benchmark for Human Activity Recognition [28], and it was collected in laboratory settings. It comprises over 114,000 video samples (each sample about 2 s; =64 h) across 120 action classes, performed by 106 subjects aged between 10 and 35 years, representing a wide range of body types and movement styles. Recordings were conducted in 32 indoor locations using Microsoft Kinect v2 sensors from three horizontal viewpoints (0°, 45°, −45°), with sensors positioned at approximately 0.8 to 2.7 m above the floor. Each sample includes synchronized RGB, depth, infrared, and 3D skeletal joint data, with 25 joints annotated per person. Data were recorded at 30 fps with a resolution of 640 × 480 pixels. The dataset covers a wide range of daily, interactive, and health-related activities. For this work, only depth and 3D joint data from single-person actions were utilized.

### 2.2. Annotation

The NTU RGB+D dataset included ground-truth data for skeleton joints and was previously annotated by the corresponding authors with activity labels. The activities involving sit, stand, and transitions were selected for this study. We used the Computer Vision Annotation Tool (CVAT) [29] to annotate skeletons and activities for the ETAP-DID.

We used the Percentage of Correct Keypoints (PCK) (see Equation (1)), which measures the percentage of joints within a certain distance (typically 15 cm), to evaluate the performance of the skeleton extraction model [30] and to assess the reliability of annotations between annotators. This metric ranges from 0 to 100%, with higher values indicating better performance.

(1) $$PCK=\frac{100}{N}\sum_{i=1}^{N}\left\{\begin{array}{cc} 1, & \mathrm{if}\;∥K_{i}-J_{i}{∥}_{2}<15\;cm\\ 0, & \mathrm{else} \end{array}\right.$$

Cohen’s Kappa (κ) (see Equation (2)), is a statistical measure used to evaluate the inter-rater agreement for activity annotations. Unlike simple percent agreement, it accounts for chance agreement. $P_{o}$ is the observed agreement between raters (i.e., the proportion of times the raters agree), and $P_{e}$ is the expected agreement by chance, calculated based on the marginal probabilities of each category.

(2) $$\kappa =\frac{P_{o}-P_{e}}{1-P_{e}}$$

Fifteen skeletal joints and several activities were annotated in the depth videos. To ensure consistency among annotators, we provided proper training and evaluation. We achieved inter-rater reliability, with average PCK scores above 85% for skeleton annotations and an average Kappa score of 90% for activity annotations. Annotated activities included walking, standing, sitting in a chair, sitting in bed, standing with a walker, standing with a walker, standing to sit, and sitting to stand.

### 2.3. Dataset Preparation

To avoid bias from differences between the two dataset setups, we took a few steps. From the different height setups in the NTU RGB+D dataset, we selected those with heights between 1.8 and 2.1 m, which are close to the camera heights in our ETAP-DID. The reduction in the reliability of body proportion at extreme heights can be avoided in this way. All camera views were included, as in the real-world ETAP-DID, which presents different camera views of the same activity. The ETAP-DID lacks ground-truth MoCap data, so manual annotations are treated as ground truth, making accurate skeleton extraction more challenging. Due to bandwidth limitations in nursing homes, fps was reduced to 6 fps.

For the HAR models, we extracted 2D skeleton joints from each dataset using a feature extraction model. The ground truth for the feature extraction model was the Mocap data in the NTU RGB+D dataset, whereas for the ETAP-DID, the ground truth was the manually annotated joints. For better model comparison, the NTU RGB+D dataset was downsampled to 6 fps to match ETAP-DID, and we used the same 15 joints across all datasets. Global 3D coordinates for each joint were computed from the depth value at the corresponding pixel and the camera intrinsics [31,32]. We considered the ETAP-DID as the older-adult dataset and the NTU RGB+D+In-lab datasets as the younger-adult dataset. In this study, we used four hours of data from older adults and six hours of data from younger adults for HAR analysis.

### 2.4. Feature Extraction

#### 2.4.1. Skeleton Extraction

The skeleton joints are extracted from depth images and used for HAR. In our previous studies [33,34], we evaluated several models on an in-lab dataset using a depth sensor and a similar setup and found that the ResNet model performed best. The skeleton extraction was implemented with ResNet models [35] and trained with L1 loss.

For the NTU RGB+D dataset, the downsampled data were used as the test set, comprising 20% of the total data. The train and validation sets were generated from the remaining 80% of the data. For the NTU RGB+D dataset, a 60:20:20 split was used for training, validation, and testing, and for ETAP-DID, an 80:10:10 split was used. L1 loss was used for backpropagation, and PCK score was used for joint similarity to the ground truth. PCK was defined as the percentage of joint predictions within 15 cm of the ground truth. The model achieved a 63% PCK score on the ETAP-DID and 88% on the NTU RGB+D dataset. The reduction in PCK score in ETAP-DID is mainly due to it being collected in the field, which introduced noisy, artifact-laden data with low visibility and distance fluctuations.

Because the extracted skeletal data originated from multiple recording sessions and sensor configurations, it was necessary to normalize the raw 3D coordinates [31]. We used a three-stage normalization. Each skeleton was translated so that the root joint (torso or hip midpoint) is at the origin.

(3) $$r=\left\{\begin{array}{cc} p_{\mathrm{torso}}, & \mathrm{if}\;\mathrm{valid},\\ \frac{1}{2}\left(p_{\mathrm{lhip}}+p_{\mathrm{rhip}}\right), & \mathrm{otherwise}. \end{array}\right.$$

All joints were translated as

(4) $${\tilde{p}}_{j}=p_{j}-r.$$

To remove viewpoint differences, skeletons were rotated into a canonical forward-facing orientation, which is based on shoulder and hip vectors [36].

(5) $$v=p_{\mathrm{rshoulder}}-p_{\mathrm{lshoulder}}\;\mathrm{or}\;p_{\mathrm{rhip}}-p_{\mathrm{lhip}}.$$

(6) $$p_{j}^{\mathrm{rot}}=R\;{\tilde{p}}_{j}.$$

To ensure consistent height across subjects, height normalization using a proxy based on femur and tibia lengths, scaling joint coordinates to an anatomical, activity-agnostic body height that remains meaningful across activities [37]. This approach is necessary because the distance between the head and ankle varies with activity (e.g., sitting) and would otherwise not scale correctly.

(7) $$h_{\mathrm{body}}=∥p_{\mathrm{hip}}^{\mathrm{rot}}-p_{\mathrm{knee}}^{\mathrm{rot}}∥+∥p_{\mathrm{knee}}^{\mathrm{rot}}-p_{\mathrm{ankle}}^{\mathrm{rot}}∥,\;p_{j}^{\mathrm{norm}}=\frac{p_{j}^{\mathrm{rot}}}{h_{\mathrm{body}}}.$$

After skeleton normalization, all joint features were standardized using z-score normalization (zero mean, unit variance) to mitigate sensor-specific biases across datasets [38].

(8) $$x^{\prime }=\frac{x-\mu_{\mathrm{train}}}{\sigma_{\mathrm{train}}}.$$

#### 2.4.2. TSFEL Features

TSFEL (Time Series Feature Extraction Library) [39] is a tool designed to automatically extract a wide range of statistical, temporal, and frequency-domain features from time series data. When applied to frame-based joint data for Human Activity Recognition, TSFEL processes joint coordinates over short time windows to generate descriptive features summarizing motion patterns within each frame. These features enable traditional machine learning models to capture important characteristics of the activity, such as variability, periodicity, and signal complexity, thereby improving frame-based classification performance.

The extraction employed a sliding window approach, with each window comprising 12 frames (equivalent to 2 s at 6 fps) and a 6-frame overlap between consecutive windows. For each window, TSFEL computed all features specified in the configuration, producing a feature matrix where each row corresponds to a window and each column to a specific extracted feature. Low-relevance TSFEL features were removed using a filter-based feature selection method (SelectKBest with ANOVA F-test), retaining only the most discriminative features; we used 250 features in our experiments.

## 3. Human Activity Recognition: Frame- and Sequence-Based Analysis

In this section, we present the conducted experiments and results on HAR using skeleton data from both datasets. We evaluate how different joint selections affect the accuracy of activity classification. Specifically, we analyze four approaches: (1) using all joints, (2) using only leg joints, (3) using non-limb joints, and (4) using only limb joints. All joints serve as a baseline, preserving body dynamics and inter-joint correlations. The leg joints are most commonly used for activities such as walking. During skeleton extraction, we observed that leg joints are the least accurately extracted due to their distance from the sensor, which motivated us to include an approach that evaluates performance without them. The final two approaches focus on understanding how limb joints affect activity recognition. The non-limb refers to the core joints, excluding the noisiest joints, which might improve robustness. The joint count affects model latency in real-world applications. Joints used in each approach are specified in Table 1. We check each frame to classify everyday activities and compare the results across these joint configurations. The activities used in the study to compare younger and older adults, as well as the activities specific to older adults, are listed in Table 2. The data were split into training and testing sets using an 80:20 ratio for all models. The split followed a subject and session-dependent approach. For each sequence, sliding windows were ensured to be in the same session and subject. The model was trained on the training set and evaluated on a held-out test set according to the predefined subject- and session-based split.

### 3.1. Metrics

We used accuracy, precision, recall, and F1-score to evaluate HAR models. Precision, recall, and F1-score are especially valuable when evaluating models on imbalanced datasets. True Positive (TP), True Negative (TN), False Positive (FP), and False Negative (FN) indicators were considered.

The confusion matrix summarizes the model’s predictions by comparing true labels against predicted labels for each class. Each cell represents the percentage of predictions for the corresponding actual class, relative to the total number of instances, providing a clearer view of class-wise accuracy. It provides insights into misclassification patterns, highlighting which actions are most often confused with one another. Each row represents the actual class, while each column shows the predicted class, allowing identification of correctly classified instances along the diagonal (higher values or brighter color) and misclassifications in the off-diagonal elements (lower values or darker color).

### 3.2. Frame-Based

We used ML models, including DT, RF, KNN, SVM, HMM, and an HMM-SVM combination, as frame-based models. These models were selected because they were used in most prior HAR work. Two input representations were evaluated: (1) raw joint coordinates extracted from depth frames and (2) TSFEL features extracted from 12-frame (2 s) sliding windows. Consequently, models trained on raw joint coordinates operate in a frame-based manner, whereas models trained on TSFEL features incorporate window-based temporal information. Joint-set strategies were evaluated on both datasets using both input representations. The model was trained on the training set, and the best hyperparameter combination was selected based on cross-validation performance. A grid search with five-fold cross-validation was performed to optimize the model’s hyperparameters.

#### 3.2.1. Decision Tree

DTs were employed here because they are simple, interpretable, and capable of handling both categorical and numerical data. Hyperparameters were optimized over the splitting criterion (Gini impurity, the maximum tree depth, the minimum tree depth, and the minimum number of samples required for node splitting and leaf nodes. To control model complexity and avoid overfitting, the maximum depth was evaluated up to 50. For TSFEL-extracted features, the optimal configuration was criterion = entropy, max_depth = 10, min_samples_split = 2, and min_samples_leaf = 1, whereas for raw joint coordinates, the best performance was achieved with criterion = entropy, max_depth = 20, min_samples_split = 2, and min_samples_leaf = 1.

The performance of the DT classifier across joint strategies, datasets, and feature types is summarized in Table 3. Frame-based raw joint features outperformed window-based TSFEL features on the ETAP-DID, except for the non-limb joint set. For the NTU RGB+D dataset, performance using raw joint features and TSFEL features remained comparable. The non-limb joint set showed better performance with TSFEL features in both datasets, whereas the leg joint set performed the worst among all sets. We used all joints with raw joint features on the ETAP-DID and the non-limb joint set with TSFEL features on set with TSFEL features for the NTU RGB+D dataset.

**Figure 2:**
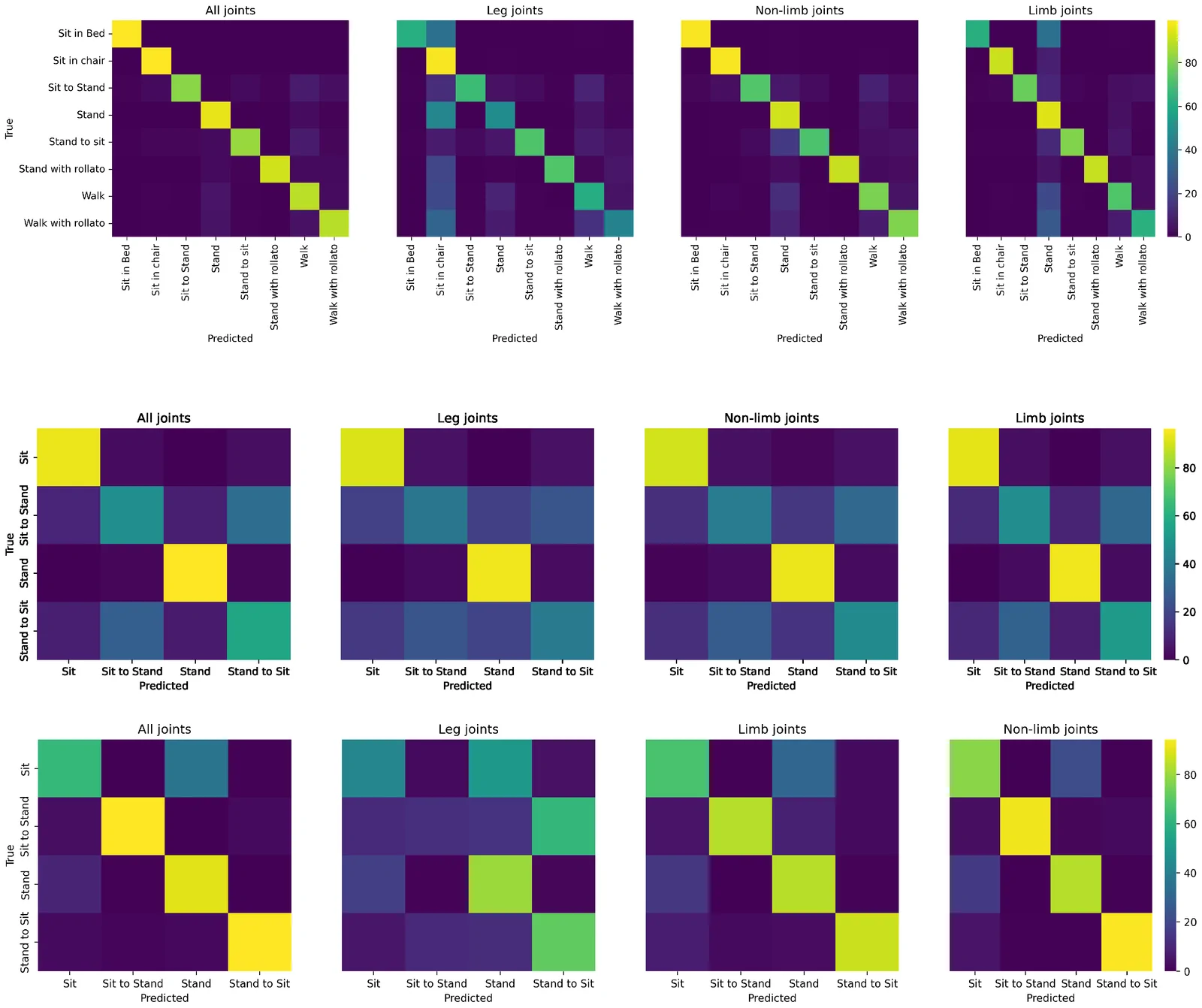
Confusion matrices for all joint strategies using DT. Top row: ETAP-DID (frame-based raw joints), middle row: NTU RGB+D dataset (frame-based raw joints), bottom row: NTU RGB+D dataset (window-based TSFEL features).

Figure 2 shows the confusion matrices for both datasets across all approaches. The confusion matrix for TSFEL features on the ETAP-DID was not shown because the F1-scores were very low, with most activities predicted as a single class. Using all joints resulted in better activity classification than using limb or non-limb joint sets. Sitting and standing activities were better classified using raw joint features, whereas transition activities performed better with TSFEL features on the NTU RGB+D dataset. For the ETAP-DID, sit- and stand-related activities were better classified than transition and walking activities.

#### 3.2.2. Random Forest

RFs are ensembles of DTs that improve classification accuracy and reduce overfitting compared to single DTs. RF combines multiple DTs, each trained on a random subset of the data and features, and aggregates their predictions to produce a robust output, even with noisy data. This approach leverages the diversity of individual trees to improve generalization. The hyperparameter search included variations in the number of estimators (50, 100, 150), maximum tree depth (None, 10, 20), minimum samples required to split a node (2, 5, 10), and minimum samples per leaf (1, 2, 4). The optimal configuration was identified as 150 estimators, no maximum depth limit, minimum samples per split = 2, and minimum samples per leaf = 1, yielding the best cross-validation performance for both datasets and feature sets.

Table 4 summarizes RF classifier performance across datasets, feature types, and joint strategies. Frame-based raw joint features consistently outperformed window-based TSFEL features on the ETAP-DID across all joint strategies, with the largest gap observed when using all joints or only leg joints. For the NTU RGB+D dataset, raw joint features generally achieved higher performance than TSFEL features, though the difference was smaller than in the ETAP-DID. The non-limb joint set showed comparable performance across the two feature types, with TSFEL features slightly outperforming raw joints in accuracy and F1-score. Across both datasets and feature types, the leg joint set performed the worst. The best overall performance was achieved using all joints with raw joint features for the ETAP-DID. For NTU RGB+D, strong performance was achieved across all joints and limb-only configurations using raw joint features, while the non-limb joint set with TSFEL features also demonstrated competitive results.

**Figure 3:**
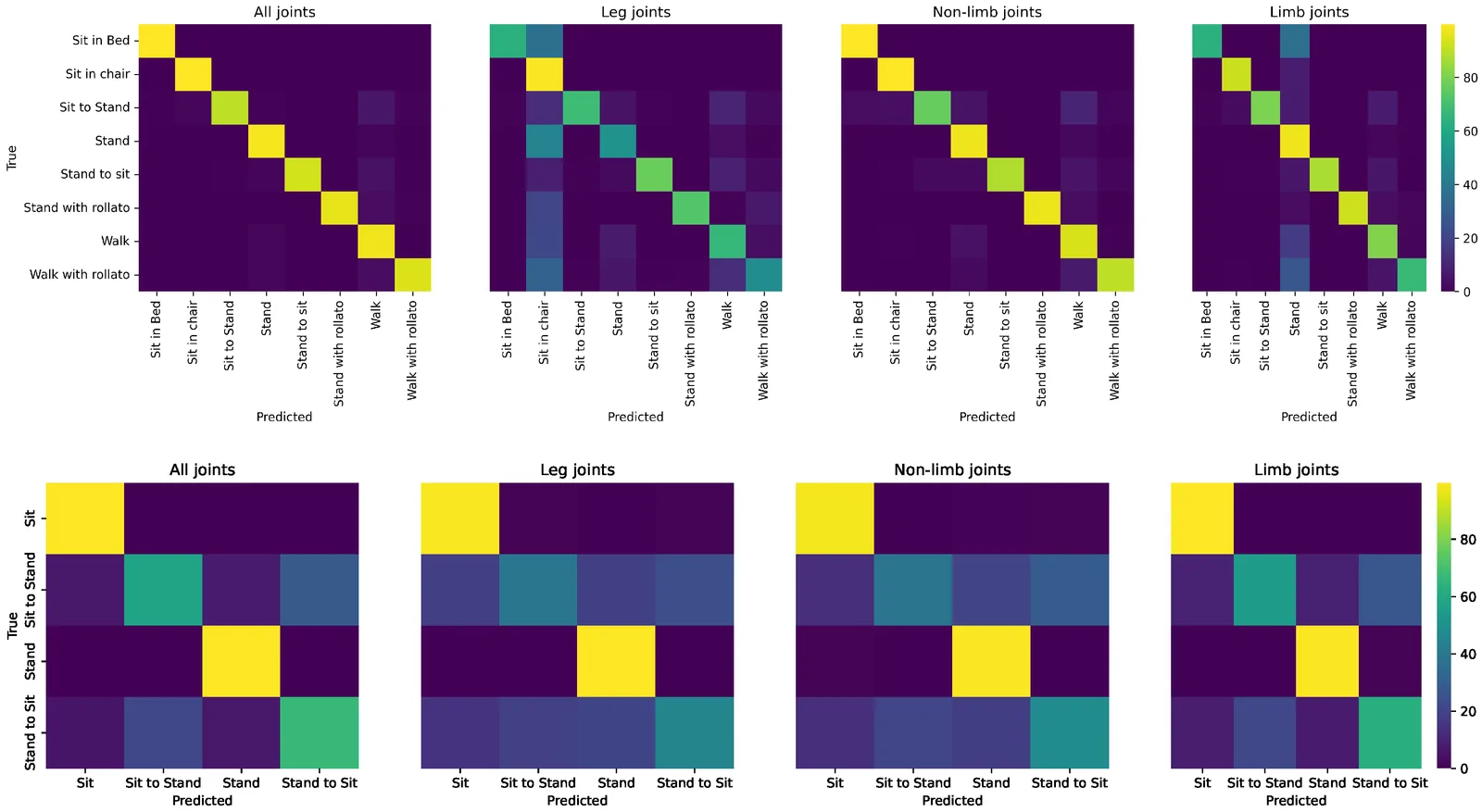
Confusion matrices for all joint strategies using RF. First row: ETAP-DID (frame-based raw joints), second row: NTU RGB+D dataset (frame-based raw joints).

The confusion matrix is given in Figure 3. In the ETAP-DID, the all-joints strategy performs best across all activities. The non-limb joint performs less well during transition activities. The limb joints strategy has more false stand predictions. The leg joint strategy performs the least, with many false predictions, during the sit-in-chair activity. For the NTU RGB+D dataset, all-joint and limb-joint strategies yield similar matrices: sit and stand are clearly classified, but transition activity predictions are confused. This trend is similar across leg and non-limb joints, with slightly lower accuracy in predicting transition activities.

##### TSFEL Window Length Comparison

The impact of window length on RF performance using window-based TSFEL features is presented in Table 5. Memory (Mem), train time (Tr.t), and inference time (In.t) are also given in the Table. The experiments were conducted only with the all-joints strategy. For the ETAP-DID, a window length of eight frames achieved better performance across all evaluation metrics than 18 frames, indicating that shorter temporal contexts are more effective at capturing discriminative patterns in this dataset. In contrast, the NTU RGB+D dataset benefited from a longer window length, with the 18-frame window achieving higher accuracy and F1-score than the eight-frame window.

##### RF Feature Importance

**Figure 4:**
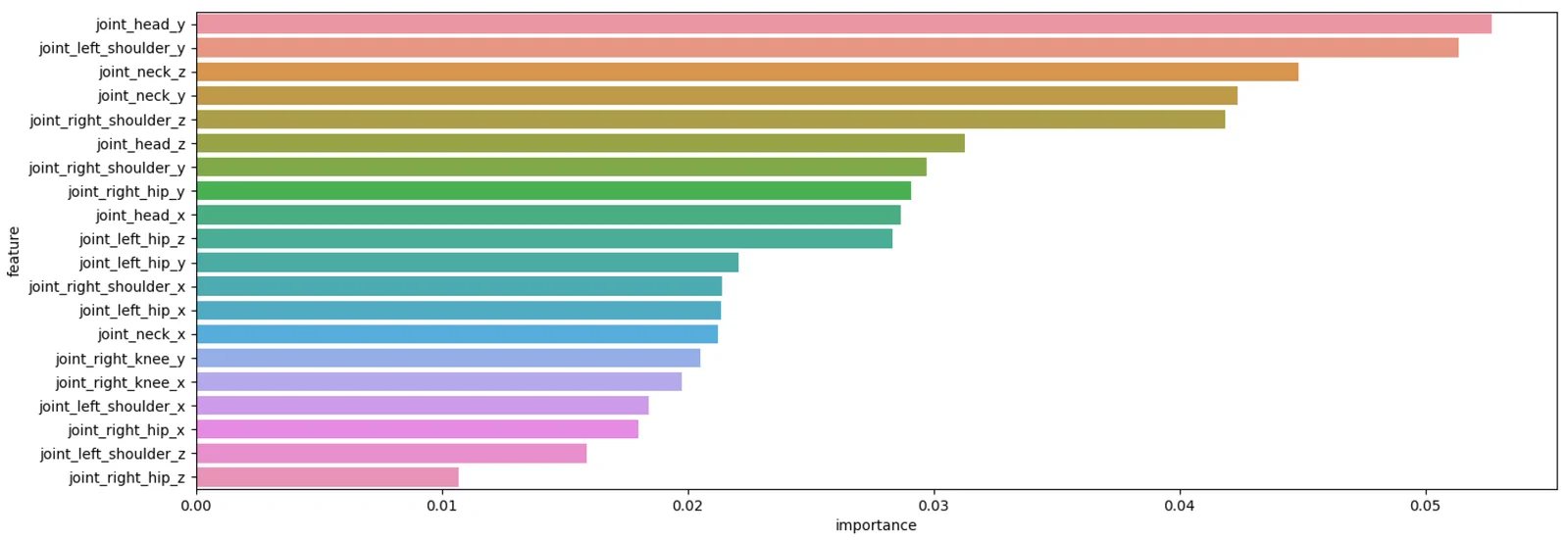
Feature importance analysis on the frame-based raw joints (first 20 features) on ETAP-DID. Different colors are used to distinguish the individual bars, while bar length represents the feature-importance value.

**Figure 5:**
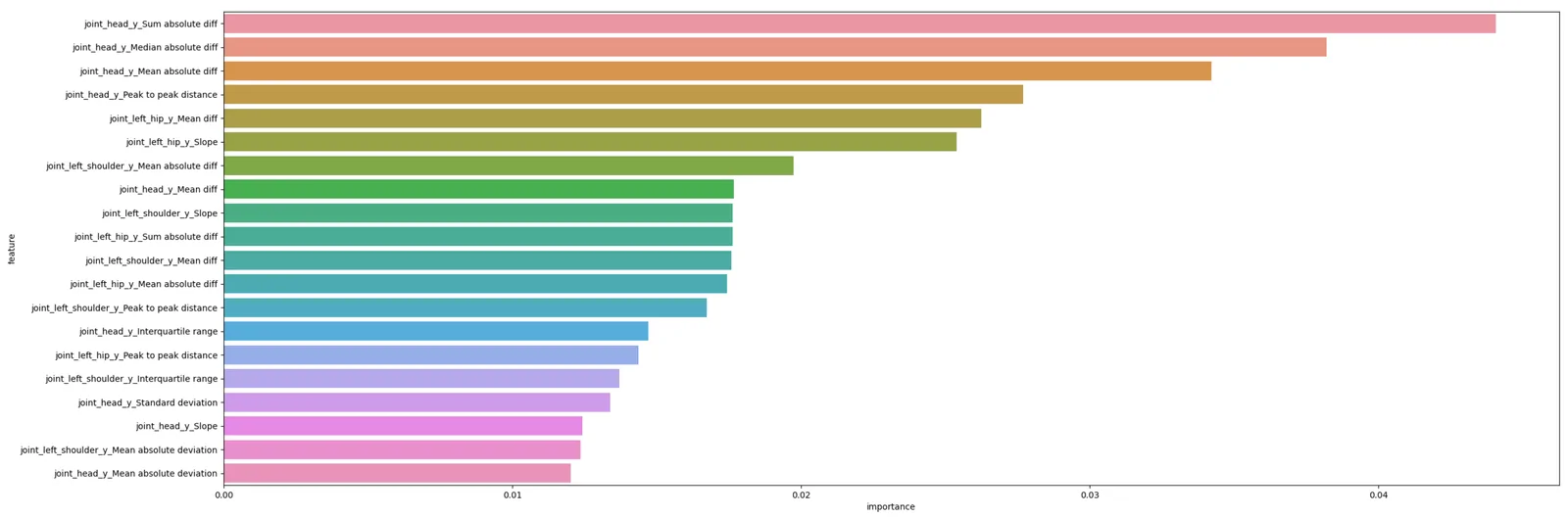
Feature importance analysis on the window-based TSFEL features (first 20 features) on ETAP-DID. Different colors are used to distinguish the individual bars, while bar length represents the feature-importance value.

Feature importance analysis of raw joints for the ETAP-DID, as shown in Figure 4, revealed that the most influential predictors were the joint positions of the head, shoulder, neck, hip, and knee. These joints are biomechanically significant; they capture both posture and motion dynamics, making them highly discriminative for human activities. Figure 5 shows the most important features from the extracted TSFEL features for the NTU dataset. The most important features included the sum, median, mean absolute differences, y-slope, and peak distance of head, hip, and shoulder movements. These capture both the magnitude of motion and the dynamic patterns (trends, variability, and periodicity), which are key discriminators among activities.

#### 3.2.3. k-Nearest Neighbors

KNN classifies data points based on the majority label of their nearest neighbors in the feature space. The algorithm relies on distance metrics to quantify similarity between samples. In this study, a distance-weighted strategy was employed, giving closer neighbors a stronger influence on predictions. The hyperparameter search explored variations in the number of neighbors (n_neighbors = 3, 5, 7, 9), weighting scheme (weights = ‘uniform’, ‘distance’), and distance metric (metric = ‘euclidean’, ‘manhattan’, ‘minkowski’). The optimal configuration was identified as nine neighbors, using distance-weighted voting with Manhattan distance, which achieved the best cross-validation performance across both datasets and feature sets.

The performance of the KNN classifier across different joint strategies, datasets, and feature types is presented in Table 6. As with the DT and RF results, frame-based raw joint features consistently outperformed window-based TSFEL features on the ETAP-DID across all joint configurations. A performance drop is observed when using TSFEL features, particularly for the leg joint set. On the NTU RGB+D dataset, raw joint features again outperformed TSFEL features across most joint strategies, although the gap was smaller than on the ETAP-DID. The non-limb joint set demonstrated comparable performance between raw joints and TSFEL features on the NTU RGB+D dataset, while raw joints clearly dominated on the ETAP-DID. Across both datasets, the leg joint configuration yielded the lowest performance, whereas using all joints or limb-only joints with raw features yielded the highest.

**Figure 6:**
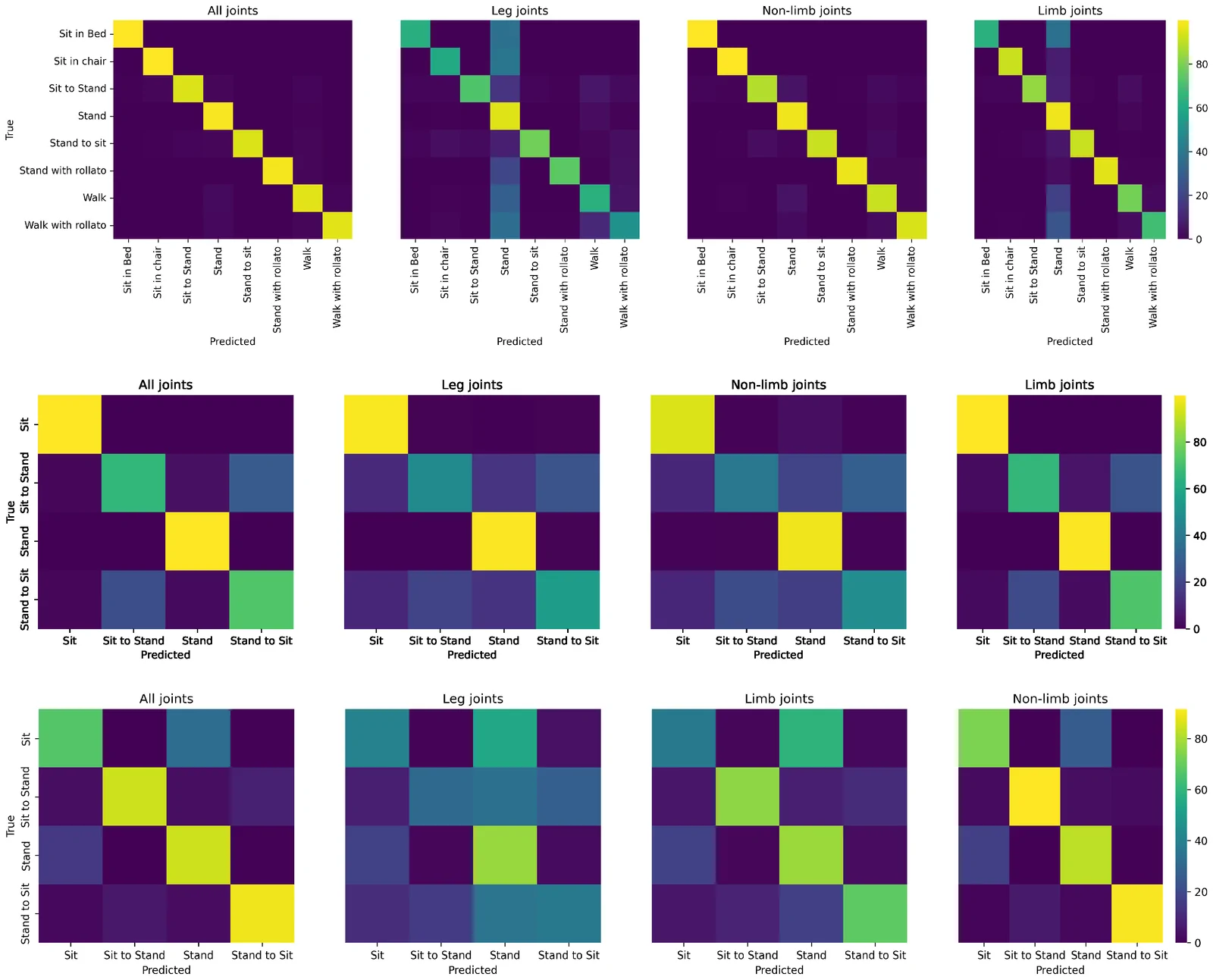
Confusion matrices for all joint strategies using KNN. Top row: ETAP-DID (frame-based raw joints), middle row: NTU RGB+D dataset (frame-based raw joints), bottom row: NTU RGB+D dataset (window-based TSFEL features).

Figure 6 shows the confusion matrices with both datasets. On the ETAP-DID with raw joint features, all-joint and non-limb strategies outperform leg- and limb-specific strategies across all activities, except for the stand activity, where many false predictions are made. The confusion matrices for the NTU RGB+D dataset show similar performance across activities for all joints and limb-joint strategies, using both frame-based raw joints and window-based TSFEL features. With TSFEL features, non-transition activities are harder to distinguish.

#### 3.2.4. Support Vector Machine

SVMs are supervised learning algorithms that identify the optimal hyperplane to separate classes in a feature space. SVMs are particularly effective for high-dimensional data and can leverage different kernel functions to model non-linear class boundaries. In this study, a hyperparameter grid search was conducted across two decision function shapes—one-vs-one (OvO) and one-vs-rest (OvR)—and four kernel functions: radial basis function (RBF), linear, polynomial, and sigmoid, yielding a total of eight hyperparameter combinations. The optimal configuration was identified as OvO with an RBF kernel for raw joints and OvO with a linear kernel for window-based TSFEL features, providing the highest classification accuracy.

**Figure 7:**
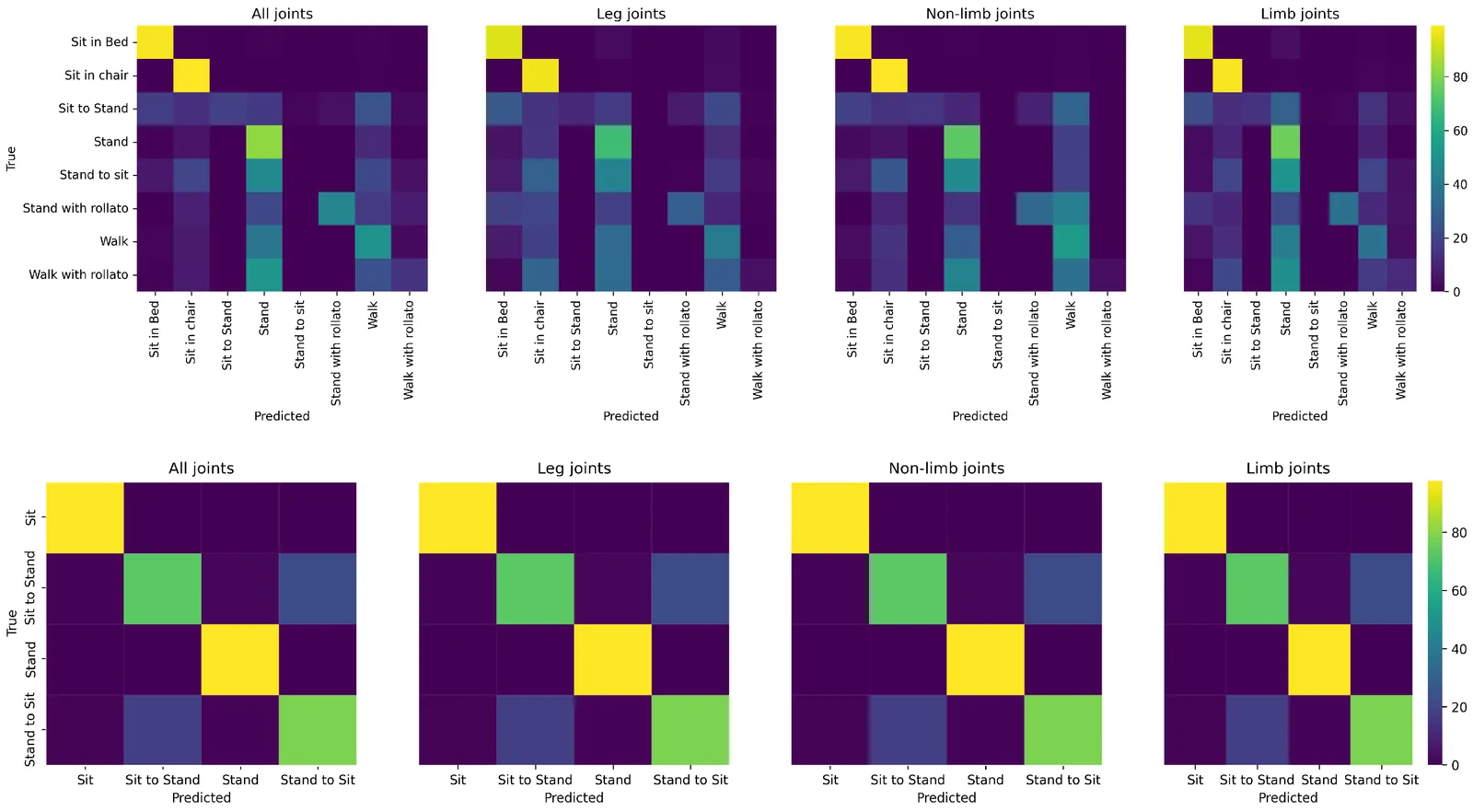
Confusion matrices for all joint strategies using SVM. First row: ETAP-DID (frame-based raw joints), second row: NTU RGB+D dataset (frame-based raw joints).

The performance of the SVM classifier using raw joint features across different joint strategies and datasets is summarized in Table 7. On the ETAP-DID, SVM performed relatively poorly across all joint strategies, with the highest accuracy with all joints (0.5423) and the lowest with limb joints only (0.3957). Precision was generally higher than accuracy, indicating that the model tended to correctly predict positive classes. For the NTU RGB+D dataset, performance was notably better, with all joints and limb-only joints achieving the highest accuracy (0.8085 and 0.8092, respectively). In comparison, non-limb and leg-only joint sets showed slightly lower performance. Figure 7 shows the confusion matrices for both datasets. For the ETAP-DID, the sit activities are well classified, but the other activities are not. The NTU RGB+D dataset shows results similar to DT and RF, in which transition activities are confused with one another.

#### 3.2.5. Hidden Markov Model

HMMs are particularly effective for sequential data and for recognizing evolving patterns, such as human activities. For the younger adult set, the number of hidden states (n_components) was between 5 and 7, whereas for the older adult set, a larger range of 8 to 16 was used to capture more activities. Different covariance types (covariance_type) were considered, including "diag", “full”, and “tied”. The maximum number of EM iterations (n_iter) was varied in the range [100, 200, 300] to ensure convergence, while the convergence tolerance (tol) was explored with [0.001, 0.01, 0.05] to control the stopping criterion. The initialization parameters (init_params) were varied among “stmc” (start probabilities, transition matrix, means, covariances) and its subsets to assess the impact of initial conditions on model performance. The optimal model was selected based on multiple random initializations and the Bayesian Information Criterion (BIC). The selected hyperparameters were n_components = 12, covariance_type = “diag”, n_iter = 300, tol = 0.01, and init_params = “stmc”. To incorporate dynamic motion information, joint velocities and accelerations were used as features alongside raw joint coordinates.

**Figure 8:**
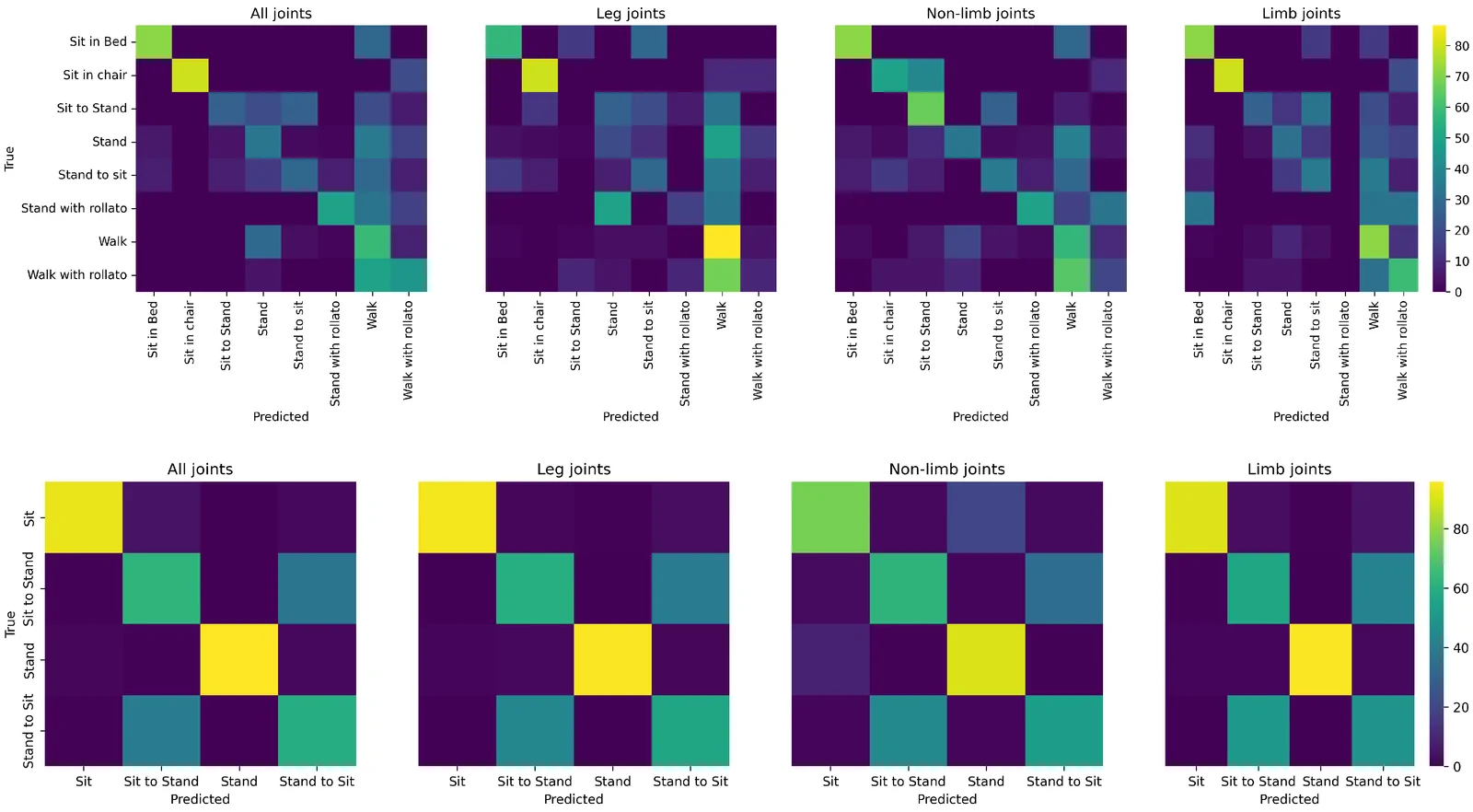
Confusion matrices for all joint strategies using HMM. First row: ETAP-DID (frame-based raw joints), second row: NTU RGB+D dataset (frame-based raw joints).

The performance of the HMM classifier across joint strategies and datasets using raw joint features is summarized in Table 8. HMM performed relatively poorly on the ETAP-DID, with all joint strategies yielding F1-score below 0.51. Among the ETAP-DID joint sets, limb joints performed the best, while leg and non-limb joints performed the worst. In contrast, performance on the NTU RGB+D dataset was substantially higher across all joint strategies, with all joints achieving the highest F1-score, followed closely by leg joints and limb joints. The non-limb joint set consistently performed lowest on NTU RGB+D but remained competitive with ETAP-DID results. These results suggest that HMM is more effective on larger datasets such as NTU RGB+D, whereas its performance is limited on smaller or more variable datasets, such as the ETAP-DID. The confusion matrices are shown in Figure 8. In the ETAP-DID, sit and walk activities are better classified than other activities. The NTU RGB+D dataset shows consistent results with previous models: non-transition activities outperform transition activities.

#### 3.2.6. Hidden Markov Model+Support Vector Model

The combined HMM-SVM approach integrates the temporal modeling strength of HMMs with the discriminative power of SVMs as observed in prior work. In this combination, HMMs model the sequential dynamics and state transitions of human activities, while SVMs classify features extracted from the HMM states, enhancing overall activity recognition performance. HMM and SVM parameters were set to the previously determined optimal hyperparameters.

**Figure 9:**
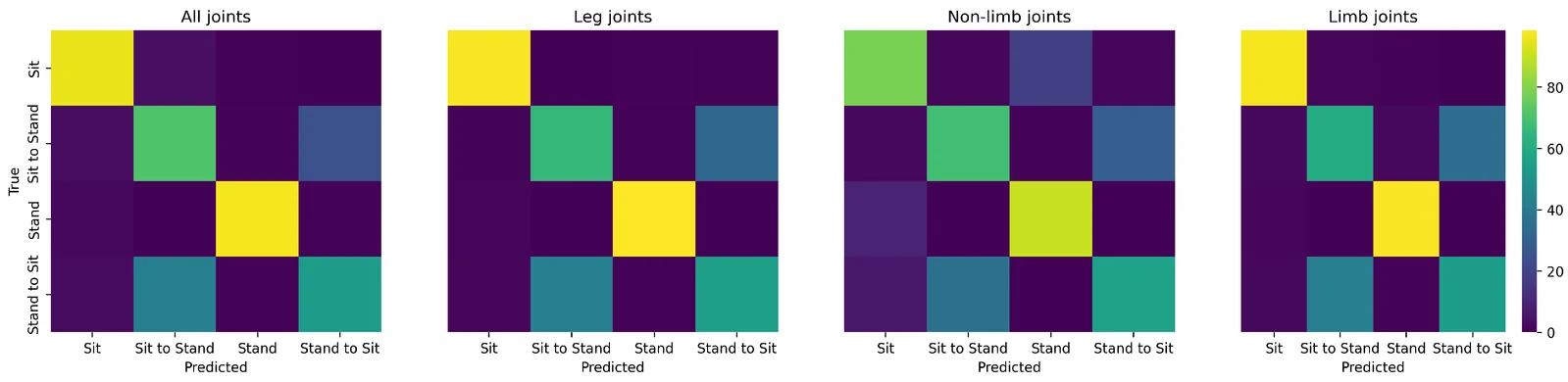
Confusion matrices for all joint strategies using HMM+SVM. First row: ETAP-DID (raw joints), second row: NTU RGB+D dataset (raw joints).

The performance of the HMM+SVM classifier across joint strategies and datasets using raw joint features is summarized in Table 9. For the ETAP-DID, overall performance was relatively low across all joint strategies, with accuracy ranging from 0.4502 for leg and non-limb joints to 0.5060 for the limb-only set. Among these, the limb-only strategy achieved the highest F1-score, although the difference was modest. In contrast, the NTU RGB+D dataset exhibited substantially higher performance, with F1-score above 0.77 across all joint strategies. The leg joint set slightly outperformed other strategies on NTU RGB+D in terms of F1-score, while the non-limb joint set showed the lowest performance among the NTU RGB+D settings. Across both datasets, the all-joint strategy did not consistently yield the best results, highlighting that performance varies with both dataset characteristics and joint selection when using HMM+SVM. The confusion matrix for the NTU RGB+D dataset is shown in Figure 9. Similar performance is observed, with more confusion during transition activities. It can also be seen that the non-limb joints perform the least, while all other strategies show very similar performance across all activities.

#### 3.2.7. Statistical Significance Between Joint Strategies

The statistical significance between joint strategies was evaluated using paired *t*-tests and Wilcoxon signed-rank tests. For the frame-based models (DT, RF, KNN) on the older adult dataset, the all-joints strategy showed greater significance (*p* < 0.001) than the leg-joints strategy. Similarly, all joints with limb joints and all joints with non-limb joints showed statistically significant (*p* < 0.001) improvements for these models. The non-limb joint set also significantly outperformed the leg joint set. Although the overall performance was lower for SVM, statistical significance comparison between leg and non-limb joint strategies showed marginal significance (*p* ≈ 0.048). The all-joints or non-limb-joints strategy yields significantly better performance than the leg-joints strategy in frame-based classifiers. For the younger adult dataset, similar trends were observed, where all joints achieved significantly higher accuracy than leg and non-limb joints. But the difference between all joints and limb joints was not significant in the KNN and SVM models. The comparison between leg and non-limb was not significant in most cases, mainly for DT and RF.

### 3.3. Sequence-Based

For sequence-based HAR, we used raw joint data sequences directly to capture long-term dependencies and motion dynamics over time. By feeding sequential joint information into models such as Long Short-Term Memory (LSTM), Temporal Convolutional Network (TCN), and attention-based Recurrent Neural Networks (ARNNs), the system can better understand complex temporal patterns and variations in human activities. This approach improves recognition accuracy by leveraging the contextual information embedded in the activity sequences.

#### 3.3.1. Long Short-Term Memory

Sequential joint information was fed into the LSTM to learn temporal patterns and contextual dependencies inherent in human activities. A two-layer LSTM network was configured with a hidden state size of 64 to capture temporal dependencies in the data while reducing overfitting, given the small dataset.

**Figure 10:**
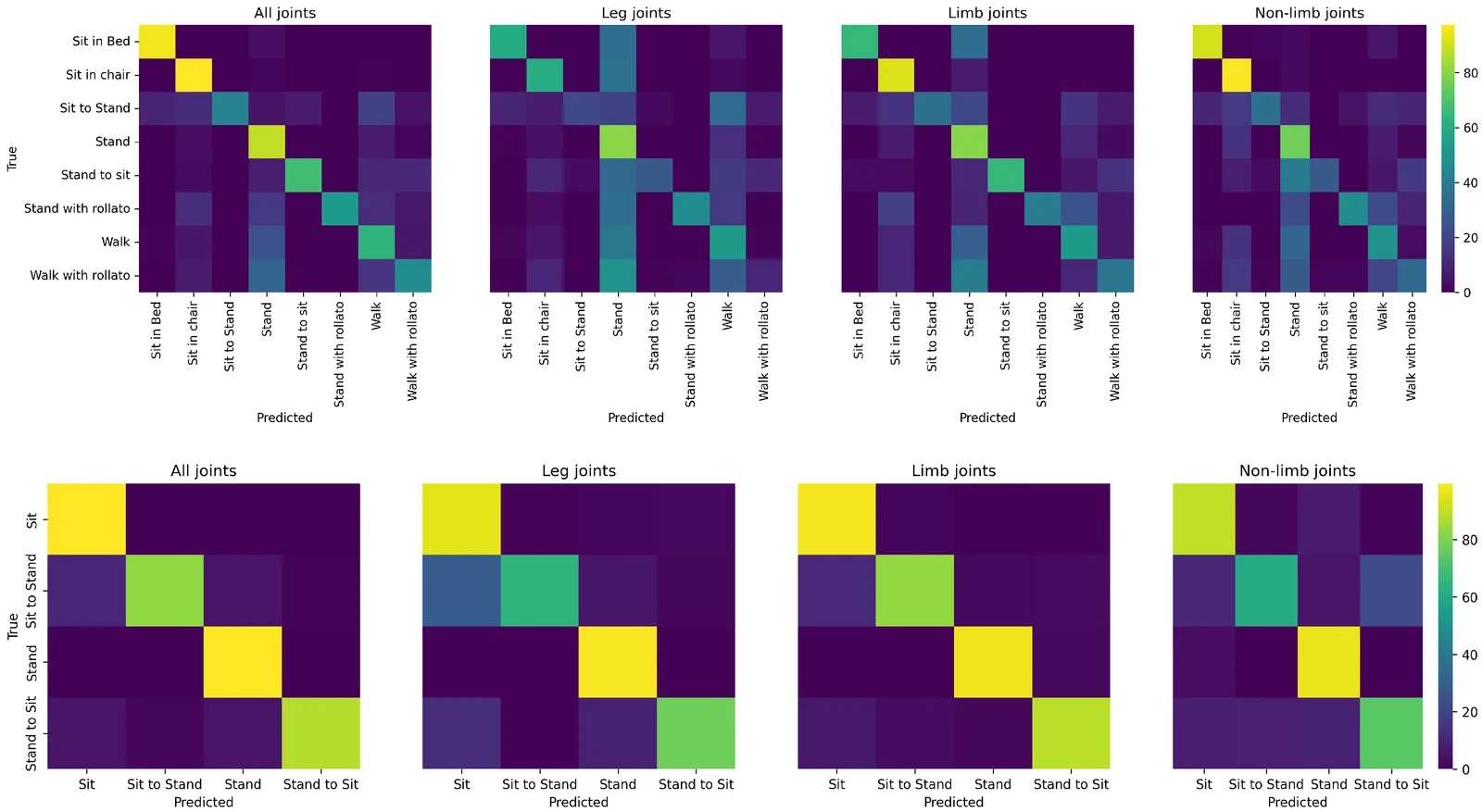
Confusion matrices for all joint strategies using LSTM. First row: ETAP-DID (window-based raw joints), second row: NTU RGB+D dataset (window-based raw joints).

The performance of the LSTM classifier using raw joint features across different joint strategies and datasets is summarized in Table 10. On the NTU RGB+D dataset, LSTM achieved very high performance across all joint strategies, with all joints yielding strong F1-scores above 0.96. Limb-only and leg-only configurations also performed well, though slightly lower than the full joint set. For the ETAP-DID, overall performance was lower, with all joints achieving an accuracy of 0.841, and the leg joint set performing the worst at 0.601. The non-limb joint set outperformed limb-only and leg-only sets for the ETAP-DID, indicating that excluding limb joints helps retain relevant information in this scenario. Figure 10 shows confusion matrices for both datasets. The all-joints strategy performs well across all activities in both datasets. Across all ETAP-DID strategies, there are more false stand predictions than false walk activity predictions. In the NTU RGB+D dataset, there is confusion between sit and transition activities, particularly with leg and non-limb joint strategies.

##### Window Length Comparison

The performance of the LSTM model using raw joint features across different window lengths is summarized in Table 11. For the ETAP-DID, a shorter window length of four frames improved overall performance compared to eight frames, achieving higher accuracy, precision, recall, and F1-score. However, this came with a modest increase in training time. In contrast, on the NTU RGB+D dataset, a longer window length of eight frames yielded superior results across all metrics compared to a four-frame window, achieving an F1-score of 0.9816. Longer windows, however, had slightly longer inference time and higher memory usage. These results indicate that the optimal window length for LSTM performance may vary across datasets, as it balances model accuracy and computational requirements.

#### 3.3.2. Temporal Convolutional Network

TCNs use dilated causal convolutions, allowing them to capture long-range temporal dependencies while preserving the chronological order of the input sequence. A TCN with two convolutional layers, each with 32 channels and a kernel size of 2, was employed to model temporal features across all cases.

The performance of the TCN classifier using raw joint features across different joint strategies and datasets is summarized in Table 12. On the NTU RGB+D dataset, the classifier performed relatively well across all joint strategies, achieving the highest accuracy with the all-joint and limb-only strategies. In contrast, performance on the ETAP-DID was lower across all joint strategies, with the leg-only and limb-only joint sets performing slightly better than all-joint or non-limb-joint strategies. The non-limb joints yielded the lowest accuracy and F1-score on the ETAP-DID, while for the NTU RGB+D dataset, all joint sets performed comparably. However, the leg-only configuration had the lowest F1-score. These results indicate that TCN is more effective on the NTU RGB+D dataset than the ETAP-DID, and that including all joints or limb joints generally provides better performance for this model.

#### 3.3.3. Attention-Based RNN

ARNNs enhance standard RNN architectures by focusing on the most relevant parts of the input sequence during prediction. The attention mechanism assigns different weights to sequence elements, enabling the network to capture important temporal cues and improve performance on complex and variable human activity sequences. For all cases, an ARNN with a hidden state size of 64 and a single linear attention layer were implemented.

**Figure 11:**
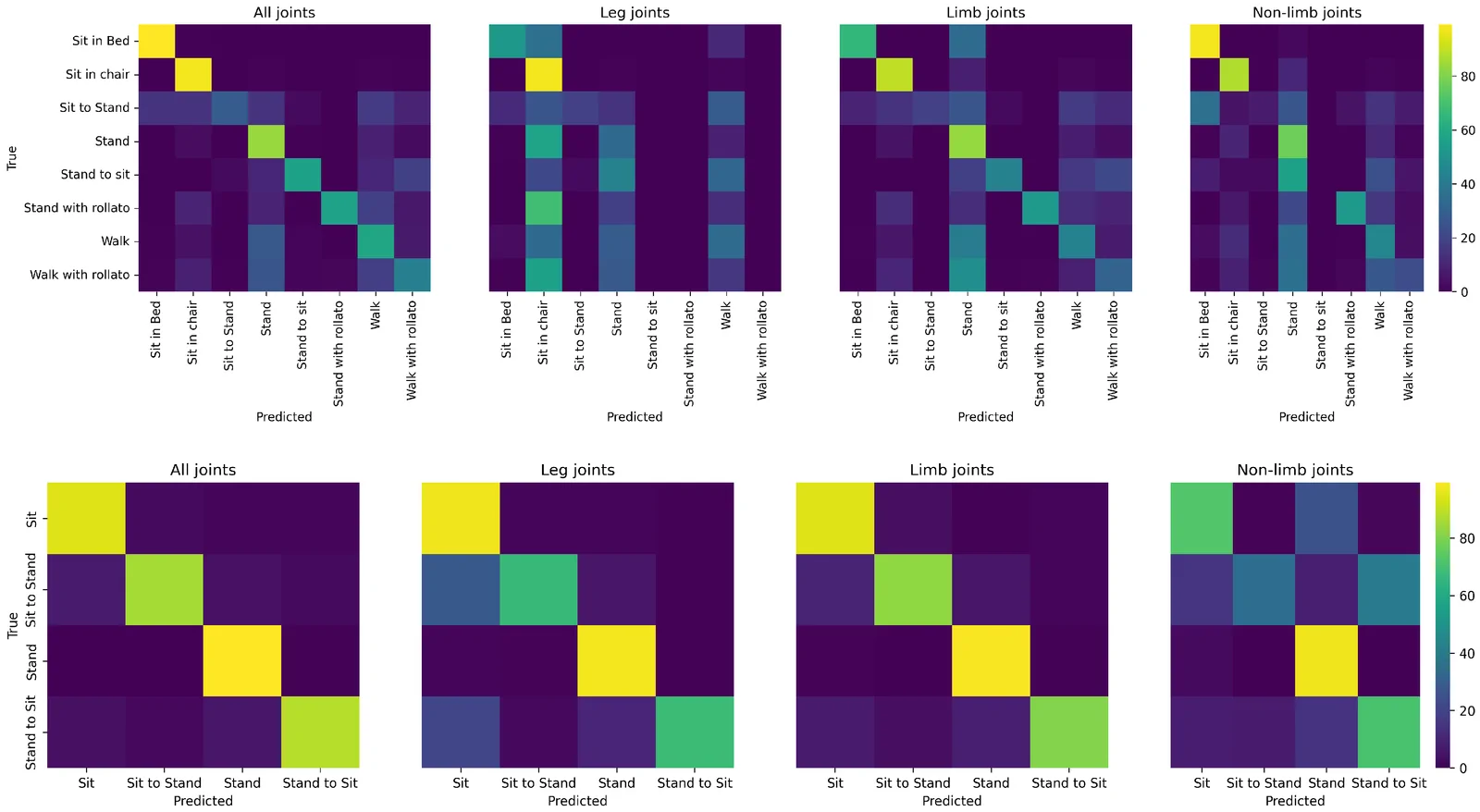
Confusion matrices for all joint strategies using ARNN. Top row: ETAP-DID (window-based raw joints), second row: NTU RGB+D dataset (window-based raw joints).

The performance of the ARNN classifier using raw joint features across different joint strategies and datasets is summarized in Table 13. For the NTU RGB+D dataset, the ARNN achieved its highest performance using all joints, with accuracy and F1-score exceeding 0.95, while limb-only and leg-only joint sets also demonstrated strong results above 0.90. In contrast, performance on the ETAP-DID was considerably lower across all joint strategies, with the highest accuracy observed for all joints and the lowest for the leg-only set. Among the joint subsets, non-limb and limb-only configurations showed moderate performance on the ETAP-DID, outperforming the leg-only set but falling short of the all-joints configuration. The confusion matrices for both datasets are shown in Figure 11. In the ETAP-DID, the leg joint strategy produced the highest number of false stand predictions. Generally, there are many false stand predictions in all the strategies. For the NTU RGB+D dataset, all joint- and limb-joint strategies show better activity classification. Still, the leg joints often predict sit activity accurately, and the non-limb strategy has more false positives and false negatives in stand and stand-to-sit activity predictions.

#### 3.3.4. Statistical Significance Between Joint Strategies

Similar to the frame-based approach, joint strategies were compared using statistical tests on sequence-based models (LSTM, TCN, ARNN). In the older adult dataset, the all-joint strategy significantly outperformed the leg, limb, and non-limb joint sets (*p* < 0.001) in LSTM and ARNN, and the difference between the limb and non-limb joint sets was small in some cases and not statistically significant. The leg joint set consistently produced significantly lower performance than limb and non-limb sets across these models (*p* < 0.001). The difference between limb and non-limb joint sets was small, being only marginally significant for LSTM (*p* ≈ 0.024) and not significant for ARNN (*p* > 0.05). For the younger adult dataset, all joints achieved the highest accuracy in most comparisons, significantly outperforming leg and non-limb joints in LSTM, TCN, and ARNN (*p* < 0.001). The leg joint set generally produced significantly lower accuracy than the other strategies. Since multiple statistical comparisons were conducted, the reported *p*-values should be interpreted with caution because family-wise error rate corrections were not applied.

### 3.4. Subject-Independent Analysis

**Figure 12:**
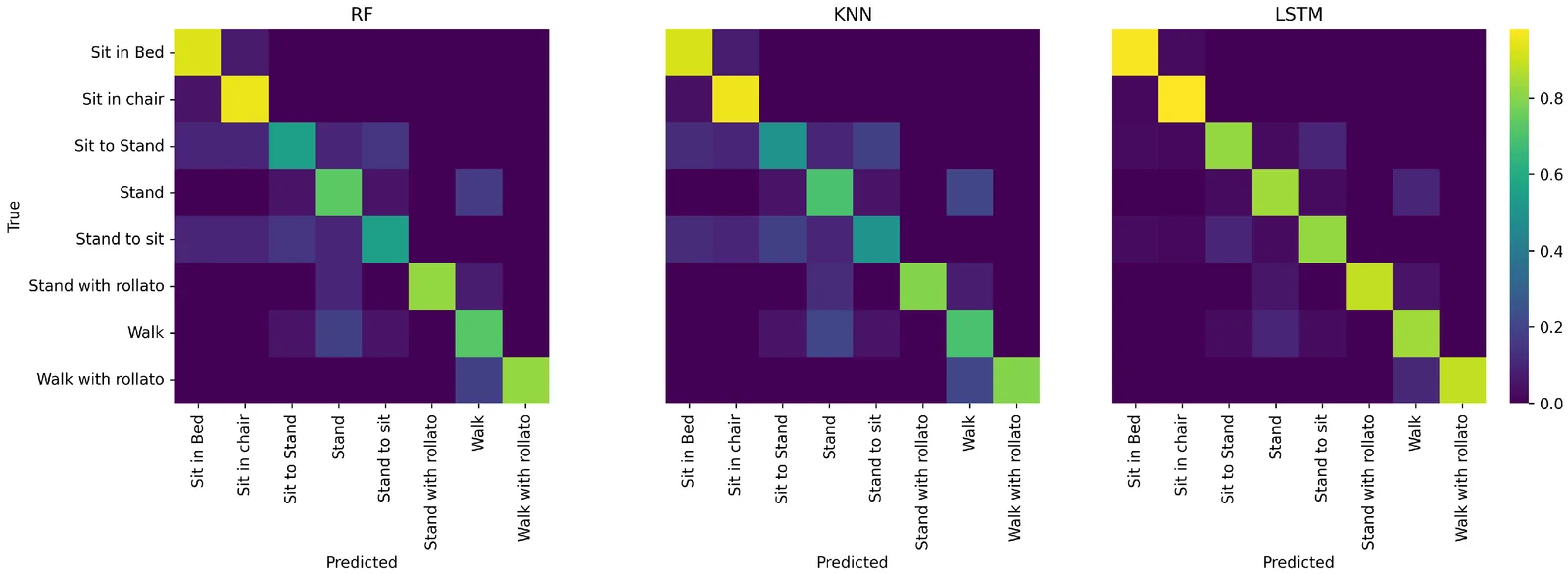
Confusion matrices for all joints strategy using subject-independent analysis using RF, KNN (frame-based raw joints), and LSTM (window-based raw joints).

As a subject-dependent evaluation was performed for HAR experiments, the reported results may overestimate generalization performance and should be interpreted accordingly. So, we analyzed the best performing models from frame and sequence-based approaches using a subject-independent split on ETAP-DID. We analyzed RF, KNN, and LSTM models, using the all joints strategy, and the performance dropped. The RF model achieved an F1-score of 0.7831 and accuracy of 0.7890, while KNN achieved an F1-score of 0.7701. The LSTM model achieved the best performance here with an F1-score of 0.8398. Figure 12 shows the confusion matrices for each model. The overall classification is comparable to previous results, with sit-related activities showing better performance.

### 3.5. Computational Performance Comparison

The computational performance of all the evaluated models using the all joints strategy with raw joints is summarized in Table 14, with DT, RF, KNN, SVM, HMM and HMM+SVM using frame-based raw joints and LSTM, TCN, and ARNN using window-based raw joints. Among classical ML approaches, DT and KNN achieved the fastest training times; DT offered extremely low inference latency, whereas KNN required longer inference time. RF showed moderate training and inference costs but higher memory usage, particularly on the ETAP-DID. In contrast, SVM and HMM+SVM were the most computationally expensive, with long training times, high memory consumption, and slow inference. In contrast, HMM alone provided a more balanced trade-off across these metrics. Among sequence models, TCN achieved fast training and inference with moderate memory requirements, outperforming recurrent models in efficiency. LSTM incurred the highest training cost despite relatively efficient inference, while ARNN showed higher overall latency than TCN. DT and TCN offered the most favorable balance between accuracy and computational efficiency. Other strategies with fewer joints slightly reduced the latency. Even a slight decrease is evident in real-time monitoring, as latency can accumulate, leading to large delays over time.

### 3.6. Discussion

The results highlight the strong influence of feature type, joint selection, and dataset characteristics on activity recognition performance. Raw joint features consistently outperformed TSFEL features on the smaller, noisier ETAP-DID. In comparison, TSFEL features provided comparable or slightly better performance on the larger NTU RGB+D dataset, particularly for non-limb joints. Using all joints generally yielded the best performance, whereas leg-only joints consistently underperformed, reflecting the importance of torso and upper-body cues. Comparable performance with non-limb joints means we obtain similar results using less noisy non-limb joints. Sit and stand activities were classified more accurately, whereas transition activities remained challenging across models. Classical models such as RF showed robust performance, while DT and KNN were more sensitive to feature choice. SVM and HMM-based methods performed poorly on the ETAP-DID but improved on NTU RGB+D, highlighting the need for larger datasets for sequential and kernel-based approaches. Sequence models (LSTM, ARNN, TCN) achieved superior performance, particularly on NTU RGB+D, with full-joint configurations and appropriately chosen window lengths. A shorter window length performed well in the window-length comparison, showing potential for real-time applications where faster detection is critical. Feature importance analyses consistently identified head, shoulders, torso, hips, knees, and ankles as key discriminative joints, aligning with biomechanical expectations. Computationally, DT and TCN were efficient for training and inference, whereas SVM was the most resource-intensive. Combining raw joint features with all joint strategy and sequence-based models yields the most reliable activity recognition, with optimal performance depending on dataset size and temporal context. Across both frame-based and sequence-based approaches, statistical testing confirmed that joint selection significantly influences activity recognition performance: the all-joint configuration generally provided the most reliable and consistent results, whereas the leg-only configuration consistently yielded the lowest performance. The performance decline under the subject-independent evaluation on ETAP-DID confirms the challenge of generalizing to unseen subjects, with the LSTM model demonstrating superior robustness compared to the traditional machine learning approaches.

Limitations of this experiment include the fact that performance varied considerably across datasets; smaller, more variable datasets, such as the ETAP-DID, yielded lower accuracy and greater confusion for transition activities, indicating limited generalizability in low-data scenarios. The accuracy of skeleton extraction limits the reduced performance on the ETAP-DID. As the models primarily use joints extracted by the skeleton extraction model, low PCK indicates inaccurate joint positions, which directly affects HAR performance. Hyperparameter optimization was limited to the all-joints approach with fewer joints; optimal hyperparameters might have differed and could have improved performance while reducing model complexity. Classical machine learning models relied heavily on raw joint positions. At the same time, handcrafted features such as TSFEL were less effective in smaller datasets, highlighting the challenge of feature engineering in low-resource settings. Sequence models were accurate on large datasets but incurred substantial computational costs and were sensitive to window length selection, which could limit their real-time applicability. These limitations can be addressed by increasing the diversity and size of training datasets, incorporating multi-view or sensor-fusion data to enrich skeletal information, leveraging data augmentation to improve generalization, refining the skeleton extraction architecture, and adopting lightweight or adaptive deep learning architectures that dynamically optimize temporal context to balance accuracy and computational efficiency.

## 4. Age Group Recognition

The AGR models were trained on a combined dataset of younger and older adults to classify individuals into age groups based on their movement patterns, distinguishing younger adults (ages 10–35, median 25 years) from older adults (ages 60 and above). The dataset prepared here combines the datasets and applies the normalization described in Section 2.3. To comprehensively evaluate age classification, multiple input representations were explored, including absolute joint positions, root-relative positions, bone length, and joint velocities, capturing both spatial and temporal aspects of the skeleton. Models were trained and evaluated using a subject-independent 80:20 split, ensuring no participants overlapped between training and test sets, to assess generalization to unseen individuals. Three models were implemented: an RF for frame-based classification with interpretable feature importance, an LSTM for modeling temporal dependencies in joint sequences, and a Spatio-Temporal Graph Convolutional Network (ST-GCN) [40] to leverage the skeletal graph structure (spatial) and temporal dependencies via graph convolutions. RF was evaluated on frame-based joints from a depth frame, while LSTM and ST-GCN were evaluated at the window-based with joints from a sequence of depth frames. The same hyperparameters as in the HAR experiments, along with a window length of 12 frames (6-frame overlap), were used here. For interpretability, RF feature importance was analyzed at the frame-based joint, while gradient-based explainability techniques, such as saliency maps [41], were applied to the LSTM and ST-GCN models to visualize the joints and frames most influential for classification.

### 4.1. Input Representations

We compute four different skeletal representations:

- Absolute joint positions
  The normalized 3D coordinates $p_{j}^{\mathrm{norm}}$
- Root-relative coordinates
  Each joint expressed relative to the torso (after normalization):
  $$p_{j}^{\mathrm{root}}=p_{j}^{\mathrm{norm}}-p_{\mathrm{root}}^{\mathrm{norm}}.$$
  This removes global translation and highlights posture and local configuration.
- Bone Length
  For each skeletal edge (u→v), we denote the parent and child joints in the kinematic tree:
  $$b_{uv}=p_{v}^{\mathrm{norm}}-p_{u}^{\mathrm{norm}}.$$
  Bone length captures intrinsic limb geometry and reduces redundancy in absolute coordinates, a representation widely used in pose-based action recognition [42]. They capture limb lengths, segment orientations, and posture patterns that often differ systematically between age groups (e.g., kyphosis, reduced hip extension, altered shoulder posture).
- Joint velocities
  Temporal motion dynamics are captured via finite differences:
  $$v_{j}\left(t\right)=p_{j}\left(t\right)-p_{j}\left(t-1\right).$$
  Velocities provide motion cues such as speed, smoothness, and acceleration trends, which are known to differ with aging—older adults often exhibit slower peak velocities, reduced joint angular speeds, and greater temporal irregularity [43]. Velocity features help models detect kinematic signatures of aging, such as slower transitions, reduced momentum generation, and compensatory limb motions.

### 4.2. Results on Combination of NTU RGB+D and ETAP-DID

Table 15 shows the performance metrics using the combined dataset. Performance metrics near 100% indicate model overestimation; sensor-specific features might have contributed in addition to joint features.

### 4.3. Results on ETAP-DID

For subsequent experiments, only the ETAP-DID was used. The older adults were in the older age group, and the staff at the facilities were in the younger age group. Hence, we used 4 h of older-adult data and 2 h of younger-adult data. The same hyperparameters and a sensor-independent 80:20 split were used. The performance of different models and input representations for age classification using the ETAP-DID is summarized in Table 16. Across all representation types, ST-GCN outperformed both the LSTM and RF models, achieving the highest F1-score. Using relative joint coordinates improved performance across all models, with ST-GCN achieving the highest F1-score of 0.8967. Velocity-based features showed similar trends, with ST-GCN again leading with an F1-score of 0.8880. Bone-based representations also yielded strong results, with ST-GCN achieving an F1-score of 0.8857, slightly outperforming LSTM and RF. Among all representations, relative joint coordinates achieved the best performance across models, followed closely by bone-based and velocity-based features.

### 4.4. Joint Importance

**Figure 13:**
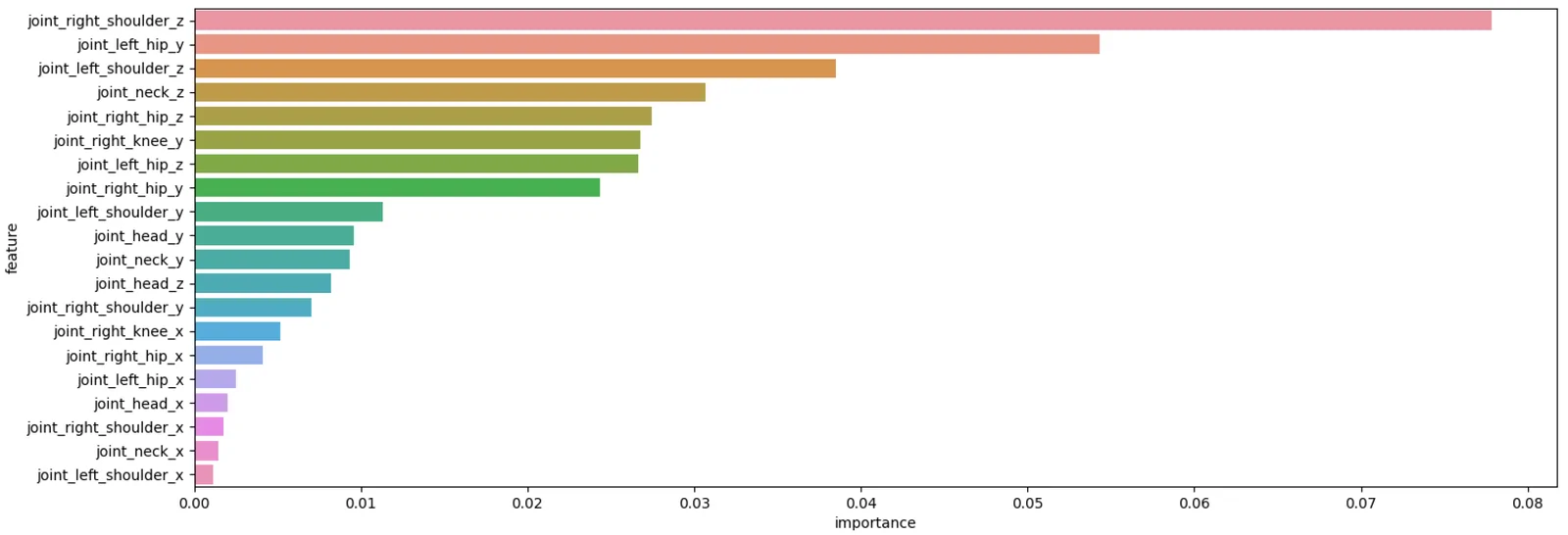
Feature importance analysis on the frame-based raw joints in AGR (first 20 features) with ETAP-DID. Different colors distinguish the individual bars, and bar length represents the feature-importance value.

**Figure 14:**
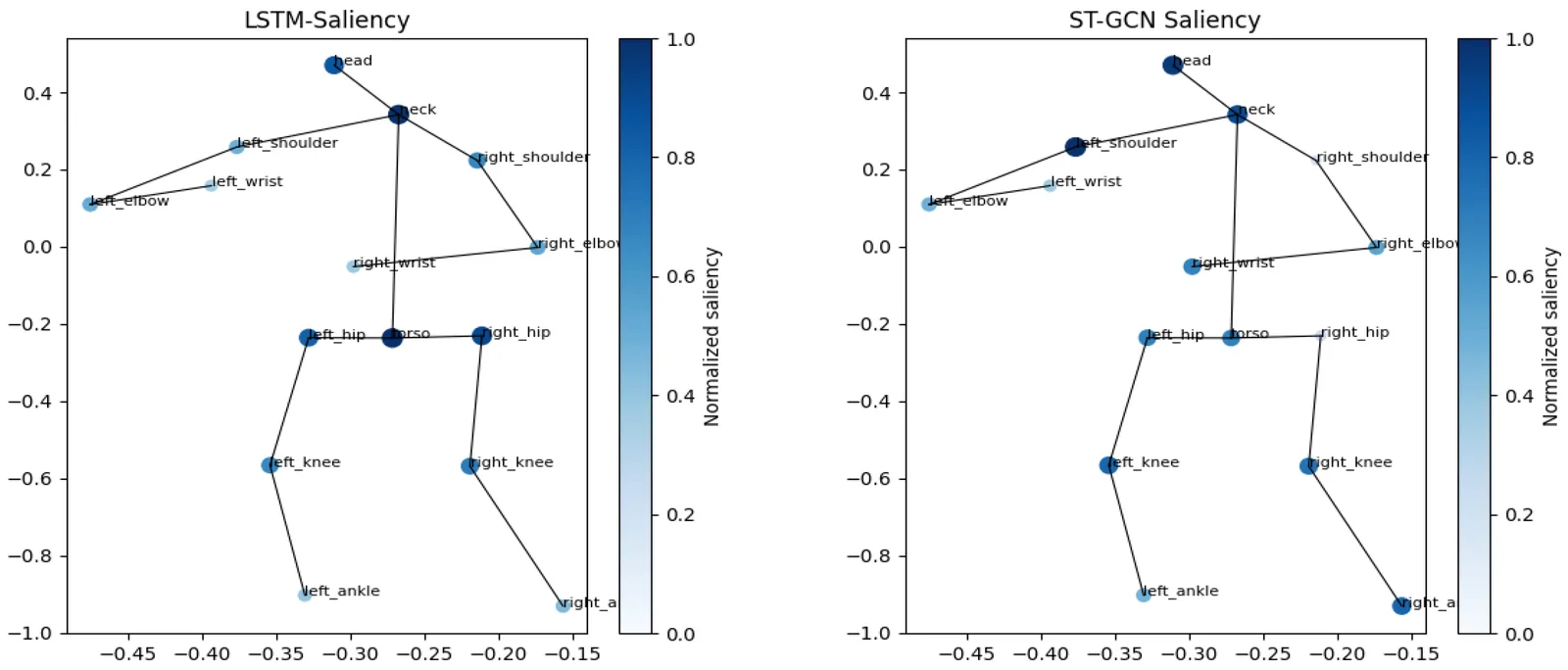
Saliency map for the LSTM and ST-GCN models highlighting the joint importance in window-based AGR.

Figure 13 shows the RF feature importance plot using absolute joints with the first 20 features. Shoulder, hip, neck, knee, and head are the most crucial features for AGR. Saliency maps with absolute representation for LSTM and ST-GCN models are given in Figure 14. The saliency maps were created by computing the absolute gradients of each joint’s xyz coordinates, averaging them over time and spatial dimensions to obtain a single importance value per joint, and then normalizing and visualizing the values as color-coded intensities on the skeleton. The head, neck, torso, shoulder, and hip are shown as the most important joints in the saliency maps.

### 4.5. Discussion

The extremely high performance observed in preliminary experiments suggests potential model overestimation, likely due to sensor-specific or environmental factors rather than true age-related motion characteristics. To address this, further experiments were limited to the ETAP-DID, in which sensing conditions were more consistent across age groups, thereby improving the reliability of the results. The results show that ST-GCN outperformed LSTM and RF models across all input representations, highlighting the importance of explicitly modeling spatiotemporal and inter-joint relationships for age classification. While RF exhibited similar performance for absolute and root-relative representations, both LSTM and ST-GCN benefited from root-relative joints. This suggests that age-related differences are primarily captured through relational posture and motion cues rather than absolute joint positions. Although ST-GCN delivered the best recognition performance, its higher computational overhead from graph construction and spatiotemporal convolutions limits its suitability for real-time deployment without hardware acceleration. Feature importance from RF and saliency maps from LSTM and ST-GCN consistently highlighted the head, neck, torso, shoulders, hips, and knees as the most informative joints. These joints are closely associated with posture, balance, and mobility, which change with age. These findings indicate that age-related movement characteristics are best captured using normalized skeletal representations and graph-based spatiotemporal models.

The limitation is that the dataset was limited to a single facility, which may restrict the models’ generalizability to other environments or populations. Although subject-independent splits were used, sensor-specific characteristics and camera placement may correlate with age labels. Future work will investigate sensor-independent evaluation and domain-adaptation techniques. To address other limitations, future work would involve incorporating larger, multi-site datasets, applying domain-adaptation or regularization techniques to reduce overfitting, and exploring multimodal inputs to capture richer age-related movement information.

## 5. Conclusions and Future Work

This study presented a comprehensive evaluation of depth-based skeleton representations for human activity and AGR across two datasets and multiple modeling paradigms. Through systematic analysis of joint configurations, feature representations, and model architectures, we demonstrated that full-body skeletal information consistently yields the highest recognition performance. In contrast, leg-only joint configurations suffer from unreliable extraction and limited discriminative power. Frame-based models benefited from handcrafted window-based TSFEL features on larger datasets, whereas raw joint representations were more effective in smaller, real-world datasets. Sequence-based models substantially outperformed classical ML approaches, particularly on the NTU RGB+D dataset, highlighting the importance of modeling temporal dynamics and inter-joint relationships. For AGR, normalized skeletal representations and ST-GCN achieved the most robust performance, with interpretable analyses revealing biomechanically meaningful joints associated with posture, balance, and mobility.

From a computational perspective, clear trade-offs between accuracy and efficiency were observed. Based on the results, TCN-based models using raw full-body joint representations are the most practical choice for real-time activity recognition, offering high accuracy, low inference latency, and stable performance across datasets. Graph-based models, while highly accurate, are better suited for offline analysis or scenarios with sufficient computational resources. The findings underscore both the potential and limitations of depth-based skeletal analysis in real-world settings, emphasizing how dataset size, sensing conditions, and representation choice affect model reliability.

Future work will focus on advancing real-time, deployment-ready skeletal analysis systems by explicitly addressing computational efficiency, latency, and scalability. Lightweight graph-based architectures and model compression techniques, such as pruning, quantization, and knowledge distillation, will be explored to reduce inference costs for ST-GCN models while preserving accuracy. Adaptive temporal modeling strategies, including variable window lengths and early-exit mechanisms, will be investigated to dynamically balance latency and recognition confidence in streaming scenarios. Sensor-independent and cross-domain evaluations will be prioritized to improve robustness against environment- and hardware-specific biases, particularly for AGR.

## Figures and Tables

**Table 1:** Joints used in the different approaches.

Approach	Joints
all joints	head, neck, torso,
	right shoulder, left shoulder,
	right hip, left hip,
	right knee, left knee,
	right ankle, left ankle,
	right elbow, left elbow,
	right wrist, left wrist
only leg joints	right knee, left knee,
	right ankle, left ankle
non-limb joints	head, neck, torso,
	right shoulder, left shoulder,
	right hip, left hip
only limb joints	right knee, left knee,
	right ankle, left ankle,
	right elbow, left elbow,
	right wrist, left wrist

**Table 2:** Activities used for each category.

Category	Activities
younger and older adults	sit
	stand
	walk
	sit to stand
	stand to sit
older adults	sit in bed
	sit in chair
	stand without aid
	stand with walker
	walk without aid
	walk with walker
	sit to stand
	stand to sit

**Table 3:** Performance comparison of DT across joint strategies and datasets (subject-dependent split) using raw joints (frame-based) and TSFEL features (window-based).

Joint Strategy	Dataset	Features	Accuracy	Precision	Recall	F1-Score
All joints	ETAP-DID	Raw joints	0.8536	0.8538	0.8536	0.8536
	ETAP-DID	TSFEL	0.5787	0.6063	0.5787	0.5458
	NTU	Raw joints	0.8344	0.8298	0.8344	0.8319
	NTU	TSFEL	0.8240	0.8232	0.8240	0.8195
Leg joints only	ETAP-DID	Raw joints	0.7037	0.7515	0.7037	0.6910
	ETAP-DID	TSFEL	0.4453	0.5611	0.4453	0.3539
	NTU	Raw joints	0.7801	0.7610	0.7801	0.7687
	NTU	TSFEL	0.6240	0.6036	0.6240	0.6044
Non-limb joints	ETAP-DID	Raw joints	0.8164	0.8159	0.8164	0.8159
	ETAP-DID	TSFEL	0.7936	0.7826	0.7936	0.7854
	NTU	Raw joints	0.7849	0.7742	0.7849	0.7790
	NTU	TSFEL	0.8363	0.8393	0.8363	0.8375
Limb joints only	ETAP-DID	Raw joints	0.8394	0.8666	0.8394	0.8407
	ETAP-DID	TSFEL	0.5795	0.6005	0.5795	0.5459
	NTU	Raw joints	0.8143	0.8113	0.8143	0.8127
	NTU	TSFEL	0.7904	0.7901	0.7904	0.7897

**Table 4:** Performance comparison of RF across joint strategies and datasets (subject-dependent split) using raw joints (frame-based) and TSFEL features (window-based).

Joint Strategy	Dataset	Features	Accuracy	Precision	Recall	F1-Score
All joints	ETAP-DID	Raw joints	0.9851	0.9852	0.9851	0.9850
	ETAP-DID	TSFEL	0.5943	0.6110	0.5943	0.5490
	NTU	Raw joints	0.8946	0.8866	0.8946	0.8894
	NTU	TSFEL	0.8240	0.8232	0.8240	0.8195
Leg joints only	ETAP-DID	Raw joints	0.7264	0.7831	0.7264	0.7170
	ETAP-DID	TSFEL	0.4538	0.5959	0.4538	0.3621
	NTU	Raw joints	0.8353	0.8134	0.8353	0.8157
	NTU	TSFEL	0.6240	0.6036	0.6240	0.6044
Non-limb joints	ETAP-DID	Raw joints	0.9685	0.9686	0.9685	0.9682
	ETAP-DID	TSFEL	0.8410	0.8350	0.8410	0.8313
	NTU	Raw joints	0.8359	0.8141	0.8359	0.8184
	NTU	TSFEL	0.8363	0.8393	0.8363	0.8375
Limb joints only	ETAP-DID	Raw joints	0.8690	0.8972	0.8690	0.8705
	ETAP-DID	TSFEL	0.5943	0.6110	0.5943	0.5490
	NTU	Raw joints	0.8814	0.8713	0.8814	0.8746
	NTU	TSFEL	0.7904	0.7901	0.7904	0.7897

**Table 5:** Performance using window-based TSFEL features using RF for different window lengths; Tr. t and In. t are reported in seconds and Mem in MB.

Dataset	Win.	Acc.	P.	R.	F1	Tr. t	In. t	Mem
ETAP-DID	8	0.5379	0.5583	0.5379	0.4921	0.3232	0.0234	4007.1406
ETAP-DID	18	0.3837	0.1472	0.3837	0.2128	0.6438	0.0711	4208.4492
NTU	8	0.8266	0.8242	0.8266	0.8253	1.1238	0.0281	4062.3828
NTU	18	0.8524	0.8485	0.8524	0.8501	1.6176	0.0648	4387.7617

**Table 6:** Performance comparison across joint strategies and datasets (subject-dependent split) using raw joints (frame-based) and TSFEL features (window-based) using KNN.

Joint Strategy	Dataset	Features	Accuracy	Precision	Recall	F1-Score
All joints	ETAP-DID	raw joints	0.9839	0.9839	0.9839	0.9839
	ETAP-DID	TSFEL	0.5617	0.5622	0.5617	0.5333
	NTU	raw joints	0.9140	0.9109	0.9140	0.9123
	NTU	TSFEL	0.7966	0.7953	0.7966	0.7953
Leg joints only	ETAP-DID	raw joints	0.7027	0.8058	0.7027	0.7116
	ETAP-DID	TSFEL	0.3615	0.4476	0.3615	0.2779
	NTU	raw joints	0.8599	0.8442	0.8599	0.8477
	NTU	TSFEL	0.5801	0.5655	0.5801	0.5646
Non-limb joints	ETAP-DID	raw joints	0.9707	0.9706	0.9707	0.9705
	ETAP-DID	TSFEL	0.7960	0.7836	0.7960	0.7870
	NTU	raw joints	0.8199	0.7999	0.8199	0.8057
	NTU	TSFEL	0.8116	0.8131	0.8116	0.8122
Limb joints only	ETAP-DID	raw joints	0.8674	0.8961	0.8674	0.8692
	ETAP-DID	TSFEL	0.5617	0.5622	0.5617	0.5333
	NTU	raw joints	0.9136	0.9097	0.9136	0.9114
	NTU	TSFEL	0.6411	0.6295	0.6411	0.6283

**Table 7:** Performance comparison across joint strategies and datasets (subject-dependent split) using raw joints (frame-based) and TSFEL features (window-based) using SVM.

Joint Strategy	Dataset	Features	Accuracy	Precision	Recall	F1-Score
All joints	ETAP-DID	raw joints	0.5423	0.7165	0.5423	0.4838
	NTU	raw joints	0.8085	0.7809	0.8085	0.7811
Leg joints only	ETAP-DID	raw joints	0.5261	0.5629	0.5261	0.4849
	NTU	raw joints	0.7388	0.7096	0.7388	0.6548
Non-limb joints	ETAP-DID	raw joints	0.5371	0.6956	0.5371	0.4757
	NTU	raw joints	0.7001	0.6619	0.7001	0.6406
Limb joints only	ETAP-DID	raw joints	0.3957	0.6501	0.3957	0.3247
	NTU	raw joints	0.8092	0.7813	0.8092	0.7826

**Table 8:** Performance comparison across joint strategies and datasets (subject-dependent split) using raw joints (frame-based) and TSFEL features (window-based) with HMM.

Joint Strategy	Dataset	Features	Accuracy	Precision	Recall	F1-Score
All joints	ETAP-DID	raw joints	0.4622	0.4771	0.4622	0.4593
	NTU	raw joints	0.8322	0.8435	0.8322	0.8373
Leg joints only	ETAP-DID	raw joints	0.4502	0.4506	0.4502	0.3986
	NTU	raw joints	0.8245	0.8371	0.8245	0.8302
Non-limb joints	ETAP-DID	raw joints	0.4502	0.4844	0.4502	0.4461
	NTU	raw joints	0.7536	0.7547	0.7536	0.7532
Limb joints only	ETAP-DID	raw joints	0.5060	0.5690	0.5060	0.5030
	NTU	raw joints	0.7977	0.8150	0.7977	0.8053

**Table 9:** Performance comparison across joint strategies and datasets (subject-dependent split) using raw joints (frame-based) with HMM+SVM.

Joint Strategy	Dataset	Features	Accuracy	Precision	Recall	F1-Score
All joints	ETAP-DID	raw joints	0.4622	0.4771	0.4622	0.4593
	NTU	raw joints	0.8504	0.8527	0.8504	0.8494
Leg joints only	ETAP-DID	raw joints	0.4502	0.4506	0.4502	0.3986
	NTU	raw joints	0.8562	0.8552	0.8562	0.8552
Non-limb joints	ETAP-DID	raw joints	0.4502	0.4844	0.4502	0.4461
	NTU	raw joints	0.7795	0.7778	0.7795	0.7779
Limb joints only	ETAP-DID	raw joints	0.5060	0.5690	0.5060	0.5030
	NTU	raw joints	0.8408	0.8386	0.8408	0.8393

**Table 10:** Performance comparison across joint strategies and datasets (subject-dependent split) using raw joints (frame-based) with LSTM.

Joint Strategy	Dataset	Features	Accuracy	Precision	Recall	F1-Score
All joints	ETAP-DID	raw joints	0.8406	0.8361	0.8406	0.8338
	NTU	raw joints	0.9638	0.9642	0.9638	0.9629
Leg joints only	ETAP-DID	raw joints	0.6009	0.6676	0.6009	0.5969
	NTU	raw joints	0.9164	0.9182	0.9164	0.9131
Non-limb joints	ETAP-DID	raw joints	0.7641	0.7508	0.7641	0.7497
	NTU	raw joints	0.8807	0.8773	0.8807	0.8775
Limb joints only	ETAP-DID	raw joints	0.7443	0.7539	0.7443	0.7382
	NTU	raw joints	0.9491	0.9495	0.9491	0.9489

**Table 11:** LSTM performance using window-based raw joints for different window lengths; Tr. t and In. t are reported in seconds and Mem in MB.

Dataset	Win.	Acc.	P.	R.	F1	Tr. t	In. t	Mem
ETAP-DID	8	0.8420	0.8372	0.8420	0.8330	1245.8924	1.2055	989.7188
ETAP-DID	4	0.8677	0.8658	0.8677	0.8658	1422.5070	0.6421	912.3359
NTU	8	0.9817	0.9818	0.9817	0.9816	1127.5128	1.2515	948.4414
NTU	4	0.9424	0.9418	0.9424	0.9403	1074.2255	0.7294	902.3359

**Table 12:** Performance comparison across joint strategies and datasets (subject-dependent split) using raw joints (window-based) with TCN.

Joint Strategy	Dataset	Features	Accuracy	Precision	Recall	F1-Score
All joints	ETAP-DID	raw joints	0.4533	0.3567	0.4533	0.3746
	NTU	raw joints	0.8430	0.8274	0.8430	0.8168
Leg joints only	ETAP-DID	raw joints	0.5226	0.4805	0.5226	0.4598
	NTU	raw joints	0.7765	0.6817	0.7765	0.7140
Non-limb joints	ETAP-DID	raw joints	0.4026	0.3131	0.4026	0.2931
	NTU	raw joints	0.7496	0.6643	0.7496	0.7032
Limb joints only	ETAP-DID	raw joints	0.5319	0.4155	0.5319	0.4455
	NTU	raw joints	0.8291	0.8090	0.8291	0.8033

**Table 13:** Performance comparison across joint strategies and datasets (subject-dependent split) using raw joints (window-based) with ARNN.

Joint Strategy	Dataset	Features	Accuracy	Precision	Recall	F1-Score
All joints	ETAP-DID	raw joints	0.8196	0.8105	0.8196	0.8121
	NTU	raw joints	0.9508	0.9503	0.9508	0.9504
Leg joints only	ETAP-DID	raw joints	0.5520	0.5168	0.5520	0.4958
	NTU	raw joints	0.9030	0.9068	0.9030	0.8991
Non-limb joints	ETAP-DID	raw joints	0.7191	0.7014	0.7191	0.7002
	NTU	raw joints	0.8018	0.8010	0.8018	0.7896
Limb joints only	ETAP-DID	raw joints	0.7245	0.7415	0.7245	0.7167
	NTU	raw joints	0.9331	0.9327	0.9331	0.9323

**Table 14:** Model comparison across different datasets using all raw joints strategy.

Model	Dataset	Train Time (s)	Inference Time (s)	Memory (MB)
DT	ETAP-DID	6.3343	0.0064	752.5572
	NTU	10.7784	0.0086	684.1981
RF	ETAP-DID	43.4258	0.3179	1812.1006
	NTU	17.9124	0.0951	703.3180
KNN	ETAP-DID	0.0258	2.7587	790.8086
	NTU	0.0224	2.9604	648.3750
SVM	ETAP-DID	220.1484	46.9227	2353.5986
	NTU	150.6142	41.0322	836.6486
HMM	ETAP-DID	76.2928	0.8290	667.1210
	NTU	82.3582	0.9290	749.0259
HMM+SVM	ETAP-DID	293.4831	47.8290	2667.1210
	NTU	232.9627	42.9290	1749.0259
LSTM	ETAP-DID	1084.5542	1.2013	919.6657
	NTU	1127.7012	1.1538	913.9140
TCN	ETAP-DID	23.0347	0.1726	934.2853
	NTU	22.8740	0.1494	938.6641
ARNN	ETAP-DID	396.0141	2.4583	939.5278
	NTU	446.0935	2.9214	972.6112

**Table 15:** Performance comparison of models and input representations for age classification using the combined dataset with subject-independent split.

Model	Rep.	Accuracy	Precision	Recall	F1-Score
RF	abs	0.9982	0.9982	0.9982	0.9982
LSTM		0.9940	0.9943	0.9940	0.9941
ST-GCN		0.9942	0.9944	0.9942	0.9943
RF	rel	0.9982	0.9982	0.9982	0.9982
LSTM		0.9953	0.9954	0.9953	0.9953
ST-GCN		0.9959	0.9960	0.9959	0.9959

**Table 16:** Performance comparison of models and input representations for age classification on the ETAP-DID with subject-independent split.

Model	Rep.	Accuracy	Precision	Recall	F1-Score
RF	abs	0.8500	0.8462	0.8478	0.8470
LSTM		0.8640	0.8613	0.8627	0.8620
ST-GCN		0.8770	0.8744	0.8758	0.8751
RF	rel	0.8820	0.8793	0.8809	0.8801
LSTM		0.8910	0.8891	0.8904	0.8897
ST-GCN		0.8980	0.8962	0.8973	0.8967
RF	bone	0.8680	0.8651	0.8667	0.8659
LSTM		0.8770	0.8750	0.8762	0.8756
ST-GCN		0.8870	0.8851	0.8863	0.8857
RF	velocity	0.8705	0.8676	0.8692	0.8684
LSTM		0.8795	0.8773	0.8786	0.8780
ST-GCN		0.8895	0.8874	0.8886	0.8880

## Data Availability

The NTU RGB+D dataset presented in the study are openly available at https://rose1.ntu.edu.sg/dataset/actionRecognition/ (accessed on 22 June 2026). The ETAP-DID cannot be shared due to the data protection and confidentiality requirements outlined in the approved study protocol and ethics agreement.

## Abbreviations

The following abbreviations are used in this manuscript:

2D	Two-Dimensional
3D	Three-Dimensional
ADL	Activities of Daily Living
ADXL210E	Analog Devices Biaxial Accelerometer Model 210E
ARNN	Attention-based Recurrent Neural Network
AGR	Age Group Recognition
CNN	Convolutional Neural Network
CVAT	Computer Vision Annotation Tool
DT	Decision Tree
ECDF	Empirical Cumulative Distribution Function
ECG	Electrocardiogram
FFT	Fast Fourier Transform
FN	False Negative
fov	Field of View
FP	False Positive
fps	Frame per Second
HAR	Human Activity Recognition
HCI	Human Computer Interface
GSV	Gait Silhouette Volume
HMM	Hidden Markov Model
IMU	Inertial Measurement Unit
KNN	k-Nearest Neighbour
LDA	Linear Discriminant Analysis
LSTM	Long Short-Term Memory
MAE	Mean Absolute Error
ML	Machine Learning
MLP	Multi-Layer Perceptron
MoCap	Motion Capture
PAMAP2	Physical Activity Monitoring Dataset 2
PCK	Percentage of Correct Keypoints
RF	Random Forest
RGB	Red, Green, Blue
RMSE	Root Mean Square Error
RNN	Recurrent Neural Network
ResNet	Residual Neural Network
SPPB	Short Physical Performance Battery
ST-GCN	Spatio-Temporal Graph Convolutional Network
SVM	Support Vector Machine
TCN	Temporal Convolutional Network
TN	True Negative
TP	True Positive
TSFEL	Time Series Feature Extraction Library
TUG	Timed-Up-and-Go
UI	User Interface
UNIBA	University of Bari
VGA	Video Graphics Array
ZVU	Zero Velocity Update

## References

[1] Cook D.J., Krishnan N.C. (2015). Activity Learning: Discovering, Recognizing, and Predicting Human Behavior from Sensor Data.

[2] Lara O.D., Labrador M.A. (2012). A survey on human activity recognition using wearable sensors. IEEE Commun. Surv. Tutor..

[3] Poppe R. (2010). A survey on vision-based human action recognition. Image Vis. Comput..

[4] Vieira E.R., Palmer R.C., Chaves P.H. (2016). Prevention of falls in older people living in the community. Br. Med. J..

[5] Chawan V.R., Huber M., Burns N., Daniel K. (2022). Person identification and Tinetti score prediction using balance parameters: A Machine Learning approach to determine fall risk. Proceedings of the 15th International Conference on Pervasive Technologies Related to Assistive Environments, Corfu, Greece, 29 June–1 July 2022.

[6] Dubois A., Bihl T., Bresciani J.P. (2021). Identifying fall risk predictors by monitoring daily activities at home using a depth sensor coupled to machine learning algorithms. Sensors.

[7] Kroll F., Löffler S., Becker I., Hofstedt P. Automatic detection of Timed-up and Go tests with IMU sensor data. Proceedings of the BIODEVICES.

[8] Hellmers S., Izadpanah B., Dasenbrock L., Diekmann R., Bauer J.M., Hein A., Fudickar S. (2018). Towards an automated unsupervised mobility assessment for older people based on inertial TUG measurements. Sensors.

[9] Pedrero-Sánchez J.F., De-Rosario-Martínez H., Medina-Ripoll E., Garrido-Jaén D., Serra-Añó P., Mollà-Casanova S., López-Pascual J. (2023). The reliability and accuracy of a fall risk assessment procedure using mobile smartphone sensors compared with a physiological profile assessment. Sensors.

[10] Guralnik J.M. (1994). Short physical performance battery assessing lower extremity and prediction of mortality and nursing home admission. J. Gerontrol..

[11] Balaji E., Brindha D., Balakrishnan R. (2020). Supervised machine learning based gait classification system for early detection and stage classification of Parkinson’s disease. Appl. Soft Comput..

[12] Cicirelli G., Impedovo D., Dentamaro V., Marani R., Pirlo G., D’Orazio T.R. (2021). Human gait analysis in neurodegenerative diseases: A review. IEEE J. Biomed. Health Inform..

[13] Wong G., Gabison S., Dolatabadi E., Evans G., Kajaks T., Holliday P., Alshaer H., Fernie G., Dutta T. (2020). Toward mitigating pressure injuries: Detecting patient orientation from vertical bed reaction forces. J. Rehabil. Assist. Technol. Eng..

[14] Patel S., Park H., Bonato P., Chan L., Rodgers M. (2012). A review of wearable sensors and systems with application in rehabilitation. J. Neuroeng. Rehabil..

[15] Hammour G.M., Mandic D.P. (2021). Hearables: Making sense from motion artefacts in ear-EEG for real-life human activity classification. Proceedings of the 2021 43rd Annual International Conference of the IEEE Engineering in Medicine & Biology Society (EMBC), Online, 1–5 November 2021.

[16] Chou E., Tan M., Zou C., Guo M., Haque A., Milstein A., Fei-Fei L. (2018). Privacy-preserving action recognition for smart hospitals using low-resolution depth images. arXiv.

[17] van der Kruk E., Strutton P., Koizia L.J., Fertleman M., Reilly P., Bull A.M. (2022). Why do older adults stand-up differently to young adults?: Investigation of compensatory movement strategies in sit-to-walk. npj Aging.

[18] Dolecka U.E., Ownsworth T., Kuys S.S. (2015). Comparison of sit-to-stand strategies used by older adults and people living with dementia. Arch. Gerontol. Geriatr..

[19] Bao L. (2004). Intille, activity recognition from user-annotated acceleration data. Proceedings of the International Conference on Pervasive 2004, Vienna, Austria, 21–23 April 2004.

[20] Benaissa B., KÖPPEN M., Yoshida K. (2018). Activity and emotion recognition for elderly health monitoring. Int. J. Affect. Eng..

[21] Ambati L.S., El-Gayar O. (2021). Human activity recognition: A comparison of machine learning approaches. J. Midwest Assoc. Inf. Syst. (JMWAIS).

[22] Gattulli V., Impedovo D., Piccinno A., Pirlo G. (2025). Human activity recognition using smartphone sensors: Focusing on fall detection with the UNIBA dataset. SN Comput. Sci..

[23] Komang M.G., Surya M.N., Ratna A.N. (2019). Human activity recognition using skeleton data and support vector machine. J. Phys. Conf. Ser..

[24] Wu H., Pan W., Xiong X., Xu S. (2014). Human activity recognition based on the combined SVM&HMM. Proceedings of the 2014 IEEE International Conference on Information and Automation (ICIA), Hulunbeir, China, 28–30 July 2014.

[25] Makihara Y., Mannami H., Yagi Y. (2010). Gait analysis of gender and age using a large-scale multi-view gait database. Proceedings of the Asian Conference on Computer Vision (ACCV), Hanoi, Vietnam, 8–12 December 2024.

[26] Gillani S.I., Azam M.A., Ehatisham-ul Haq M. (2020). Age estimation and gender classification based on human gait analysis. Proceedings of the 2020 International Conference on Emerging Trends in Smart Technologies (ICETST), Karachi, Pakistan, 26–27 March 2020.

[27] Paul R.E., Kock P., Hartmann Y., Ball E., Seibert K., Liu H., Schultz T. (2025). Longitudinal data acquisition for AI services in long-term care facilities for older adults. Proceedings of the 18th International Joint Conference on Biomedical Engineering Systems and Technologies—Scale-IT-Up, Porto, Portugal, 19–21 February 2025.

[28] Liu J., Shahroudy A., Perez M., Wang G., Duan L.Y., Kot A.C. (2019). NTU RGB+D 120: A large-scale benchmark for 3D human activity understanding. IEEE Trans. Pattern Anal. Mach. Intell..

[29] CVAT.ai Corporation (2023). Computer Vision Annotation Tool (CVAT), Version 2.2.0.

[30] Zheng C., Wu W., Chen C., Yang T., Zhu S., Shen J., Kehtarnavaz N., Shah M. (2022). Deep learning-based human pose estimation: A survey. arXiv.

[31] Shotton J., Fitzgibbon A., Cook M., Sharp T., Finocchio M., Moore R., Kipman A., Blake A. (2011). Real-time human pose recognition in parts from single depth images. Proceedings of the IEEE Conference on Computer Vision and Pattern Recognition (CVPR), Colorado Springs, CO, USA, 20–25 June 2011.

[32] Zhang Z. (2012). Microsoft Kinect sensor and its effect. IEEE Multimed..

[33] Hartmann Y., Klöckner J., Deichsel L., Paul R., Schultz T. (2024). Gait parameter estimation from a single privacy preserving depth sensor. Proceedings of the 17th International Joint Conference on Biomedical Engineering Systems and Technologies–Volume 1: BIOSIGNALS.

[34] Hartmann Y., Paul R.E., Klöckner J., Deichsel L., Schultz T. (2025). Gait parameter estimation from a single depth sensor. J. Smart Cities Soc..

[35] Sun X., Shang J., Liang S., Wei Y. Compositional human pose regression. Proceedings of the IEEE International Conference on Computer Vision (ICCV).

[36] Moon G., Chang J.Y., Lee K.M. Camera distance-aware top-down approach for 3D multi-person pose estimation from a single RGB image. Proceedings of the IEEE/CVF International Conference on Computer Vision (ICCV).

[37] Boyer K.A., Johnson R.T., Banks J.J., Jewell C., Hafer J.F. (2017). Systematic review and meta-analysis of gait mechanics in young and older adults. Exp. Gerontol..

[38] Ioffe S., Szegedy C. Batch normalization: Accelerating deep network training by reducing internal covariate shift. Proceedings of the International Conference on Machine Learning. pmlr.

[39] Barandas M., Folgado D., Fernandes L., Santos S., Abreu M., Bota P., Liu H., Schultz T., Gamboa H. (2020). TSFEL: Time series feature extraction library. SoftwareX.

[40] Yan S., Xiong Y., Lin D. Spatial temporal graph convolutional networks for skeleton-based action recognition. Proceedings of the Association for the Advancement of Artificial Intelligence (AAAI) Conference on Artificial Intelligence.

[41] Simonyan K., Vedaldi A., Zisserman A. (2013). Deep inside convolutional networks: Visualising image classification models and saliency maps. arXiv.

[42] Ke Q., Bennamoun M., An S., Sohel F., Boussaid F. A new representation of skeleton sequences for 3D action recognition. Proceedings of the IEEE Conference on Computer Vision and Pattern Recognition (CVPR).

[43] Zhang P., Lan C., Xing J., Zeng W., Xue J., Zheng N. (2019). View adaptive neural networks for high performance skeleton-based human action recognition. IEEE Trans. Pattern Anal. Mach. Intell..

